# Mitophagy is required for brown adipose tissue mitochondrial homeostasis during cold challenge

**DOI:** 10.1038/s41598-018-26394-5

**Published:** 2018-05-29

**Authors:** Yuan Lu, Hisashi Fujioka, Dinesh Joshi, Qiaoyuan Li, Panjamaporn Sangwung, Paishiun Hsieh, Jiyun Zhu, Jose Torio, David Sweet, Lan Wang, Shing Yan Chiu, Colleen Croniger, Xudong Liao, Mukesh K. Jain

**Affiliations:** 1 Cardiovascular Research Institute, Department of Medicine, Case Western Reserve University School of Medicine and Harrington Heart and Vascular Institute, University Hospitals Cleveland Medical Center, Cleveland, Ohio, USA; 2 Electron Microscopy Facility, Case Western Reserve University, Cleveland, Ohio, USA; 3 Department of Neuroscience, University of Wisconsin School of Medicine and Public Health, Madison, Wisconsin USA; 4 Department of Cardiology, Beijing Anzhen Hospital, Beijing Capital Medical University, Beijing, China; 5 Illinois Mathematics and Science Academy, Aurora, IL USA; 6 Department of Nutrition, Case Western Reserve University School of Medicine, Cleveland, Ohio, USA

## Abstract

Brown adipose tissue (BAT) is a specialized thermogenic organ in mammals. The ability of BAT mitochondria to generate heat in response to cold-challenge to maintain core body temperature is essential for organismal survival. While cold activated BAT mitochondrial biogenesis is recognized as critical for thermogenic adaptation, the contribution of mitochondrial quality control to this process remains unclear. Here, we show mitophagy is required for brown adipocyte mitochondrial homeostasis during thermogenic adaptation. Mitophagy is significantly increased in BAT from cold-challenged mice (4 °C) and in β-agonist treated brown adipocytes. Blockade of mitophagy compromises brown adipocytes mitochondrial oxidative phosphorylation (OX-PHOS) capacity, as well as BAT mitochondrial integrity. Mechanistically, cold-challenge induction of BAT mitophagy is UCP1-dependent. Furthermore, our results indicate that mitophagy coordinates with mitochondrial biogenesis, maintaining activated BAT mitochondrial homeostasis. Collectively, our *in vivo* and *in vitro* findings identify mitophagy as critical for brown adipocyte mitochondrial homeostasis during cold adaptation.

## Introduction

Brown adipose tissue (BAT) evolved as a specialized thermogenic organ in modern eutherian mammals^1,2^. During cold-challenge, BAT metabolizes nutrients (glucose and fatty acids) through non-shivering thermogenesis (NST), a critical adaptation that helps maintain homeothermy^3^. In addition to its thermogenic function, activated BAT also contributes significantly to systemic metabolic homeostasis^4^, implicating BAT in the management of metabolic diseases, such as diabetes and obesity^5–11^. Brown adipocytes (BA), the functioning unit of BAT, are robustly endowed with mitochondria that are central to their thermogenic and metabolic functions^12^. In BA mitochondria, energy generated from nutrients is first stored as proton motive potential across the mitochondrial inner membrane, then converted directly into heat by uncoupling protein 1 (UCP1)-mediated proton leak^2^. The heat is then distributed by blood flow to help maintain core body temperature. Given the centrality of mitochondria to BA function, control of mitochondrial health is of paramount importance and processes such as mitochondrial biogenesis, repair, and removal (quality control) are likely essential for optimal BA mitochondrial homeostasis^13^. Indeed, previous studies show that in response to cold-challenge, mitochondrial biogenesis is induced and required for optimal BAT NST function^14^. However, whether mitochondrial quality control has a role in BA thermogenesis remains unknown. In the present study, we reveal that mitochondrial selective autophagy (termed mitophagy) is necessary for maintaining BAT mitochondrial integrality and optimal BAT thermogenesis function. Our data also indicates that mitophagy acts in concert with mitochondrial biogenesis to maintain mitochondrial homeostasis in activated BAT.

## Results and Discussion

### Enhanced brown adipose tissue mitophagy during cold-challenge

**Figure 1:**
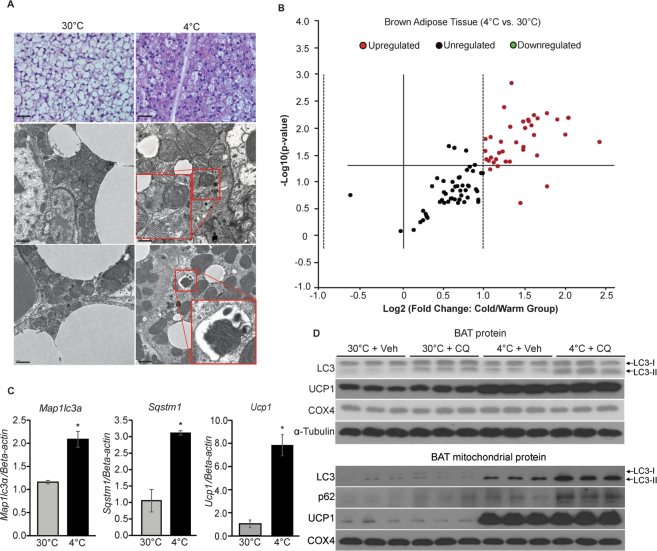
Increased BAT mitophagy upon cold-challenge. (**A**) Representative histology and EM pictures of cold-challenged BAT (n = 4 mice in each group). EM pictures show mitophagosomes (outlined by the red boxes and amplified in the insets). Scale bars: 40 μm (upper panels); 1 μm (lower panels). (**B**) Volcano plot of autophagy gene profile qPCR comparing 7d 4 °C-challenged (n = 3 mice) to 30 °C-acclimated (n = 4 mice) mice BAT. Red dots in the upper right panel are the genes significantly increased by 2 fold (p < 0.05) in 4 °C-challenged BAT. The data was analyzed by GeneGlobe Data Analysis Center (Qiagen). (**C**) BAT *Map1lc3a*, *Sqstm1* and *Ucp1* mRNA levels. n = 5 mice per group. Data are expressed as mean ± SEM as compared to BAT mRNA extracts for thermoneutrality. *p < 0.05 by two-tailed Student’s t-test. (**D**) Western blots of autophagy markers LC3 and p62; UCP1 and Cox4 protein levels in BAT tissue protein extracts (upper panel) and mitochondrial protein extracts (lower panel) from 7d 4 °C-challenged mice and 30 °C-acclimated mice with Veh (0.9% Saline) or CQ treatment (n = 3 mice in each group). Veh: vehicle; CQ: chloroquine.

To explore how activated BAT mitochondria maintain homeostasis during cold-challenge, we first obtained electron microscopic (EM) images to evaluate BAT mitochondrial ultrastructure from mice subjected to 4 °C (cold-challenge) vs. 30 °C (thermoneuturality) for 7 days^2,15^. EM images revealed the presence of numerous mitophagosomes in cold-challenged mouse BAT (representative pictures are shown in Fig. 1A outlined by the red boxes). Consistent with the enhanced mitophagy seen on EM, analysis of an Quantitative PCR array of 84 autophagy related genes revealed that ~50% of those were significantly increased by more than 2 fold in 7-day cold-challenged BAT (Fig. 1B and Table 1). mRNA expression levels of mammalian autophagic membrane markers microtubule-associated protein light chain 3 (*Map1lc3a*) and ubiquitin-interacting protein p62 (*Sqstm1*)^16–18^ were significantly increased by qPCR concordant with increased BA specific mitochondrial inner membrane protein uncoupling protein 1 (*Ucp1*), validating results from the autophagy array (Fig. 1C). In parallel, we observed significantly increased levels of mitochondrially encoded DNA cytochrome c oxidase I (mt-Co1), cytochrome c oxidase II (mt-Co2) and ATP Synthase 6 (mt-Atp6) (Supplementary Fig. S1). Next, we compared expression of autophagic markers microtubule-associated protein light chain 3 (LC3) and ubiquitin-interacting protein p62 (p62) in BAT protein extracts^18,19^. Because the measurements of protein expression are steady-state methods and autophagy is highly dynamic, we utilized the lysosomotropic reagent chloroquine (CQ) to evaluate autophagy flux, which is a preferred way to study autophagy pathway activation^16^. The autophagic process includes autophagosome formation, autolysosome conversion, degradation of autophagic substances inside the autolysosome and the subsequent release of the breakdown products. Lysosomotropic reagents (e.g., CQ and Bafilomycin A) either inhibit downstream acidification inside the lysosome or inhibit autophagosome-lysosome fusion, allowing the observation of substrate entry into the autophagy pathway and subsequent autophagosome accumulation without contribution from the exit of the pathway^16^. First, we evaluated the drug effect of CQ on BAT mitochondria by administering CQ at 30 mg/kg/day for 7 days to mice at thermoneutrality. We observed no significant changes in BA mitochondrial ultrastructure or mouse core body temperature after CQ treatment (Supplementary Fig. S2A and B). This finding is in line with low levels of autophagic flux in BAT of mice at thermoneutrality. Next, we treated mice with Vehicle (Veh, 0.9% saline) or CQ (30 mg/kg/day) for 7 days and compared autophagy marker LC3 protein levels from mice subjected to cold-challenge vs. thermoneutrality. Compared to BAT from 30 °C, expression of membrane-bound LC3-phosphatidylethanolamine (PE) conjugate (LC3-II) was higher in 4 °C-challenged BAT tissue protein extracts, as well as in mitochondrial protein extracts. Further, both LC3-II and p62 expression were significantly elevated in mitochondrial protein extracts from chloroquine-treated mice, indicating increased mitophagy flux (Fig. 1D). In conclusion, these results show significantly increased mitophagy in cold-challenged BAT.

**Table 1:** Quantitative PCR array of autopahgy related genes from 7d cold-challenged BAT (n = 3 mice) vs. BAT at thermoneutrality (n = 4 mice).

Position	Gene Symbol	Fold Regulation	p-value
A01	Akt1	3.44	0.005
A02	Ambra1	4.40	0.005
A03	App	2.47	0.014
A04	Atg10	3.21	0.231
A05	Atg12	2.20	0.113
A06	Atg16l1	2.66	0.029
A07	Atg16l2	2.96	0.001
A09	Atg4a	2.38	0.027
A10	Atg4b	2.12	0.050
A11	Atg4c	2.27	0.051
A12	Atg4d	3.52	0.006
B02	Atg7	3.29	0.018
B03	Atg9a	3.70	0.023
B04	Atg9b	2.70	0.019
B05	Bad	3.28	0.005
B06	Bak1	4.03	0.003
B07	Bax	4.73	0.012
B08	Bcl2	2.01	0.031
B10	Becn1	3.34	0.009
B11	Bid	3.61	0.011
C03	Cdkn1b	2.03	0.161
C04	Cdkn2a	2.46	0.052
C08	Ctss	2.06	0.044
C09	Cxcr4	2.48	0.030
C10	Dapk1	3.61	0.005
C12	Dram2	2.04	0.095
D03	Esr1	2.36	0.021
D04	Fadd	2.44	0.034
D05	Fas	2.03	0.081
D06	Gaa	3.29	0.018
D08	Gabarapl1	2.62	0.044
D11	Hdac6	3.42	0.005
D12	Hgs	4.86	0.005
E05	Igf1	2.77	0.005
E08	Lamp1	2.18	0.121
E09	Map1lc3a	4.86	0.005
E12	Mapk8	3.08	0.021
F05	Pik3cg	4.05	0.105
F07	Prkaa1	6.36	0.014
F08	Pten	2.58	0.037
F10	Rb1	2.54	0.038
G01	Snca	4.86	0.005
G02	Sqstm1	2.22	0.176
G04	Tgm2	2.81	0.035
G05	Tmem74	2.16	0.042
G06	Tnf	2.22	0.200
G07	Tnfsf10	2.36	0.011
G09	Ulk1	2.74	0.022
G10	Ulk2	2.30	0.054
G11	Uvrag	2.93	0.007
G12	Wipi1	3.71	0.005

We next questioned whether mitophagy played a role in skeletal muscle (SKM) mediated shivering thermogenesis, in addition to BAT mediated non-shivering thermogenesis (NST). Upon cold-challenge, mammals utilize both shivering and NST mechanisms to maintain euthermic body temperature. When mice are acutely transferred to 4 °C, SKM shivering generates extra heat to defend body temperature. Enhanced BAT recruitment and NST result in the disappearance of thermogenesis from SKM shivering in 2–3 weeks. At around 1 week of cold-challenge, the recruitment of BAT NST reaches sub-optimal level and thermogenesis from SKM shivering plateaus^2,20^. Thus, we investigated if mitophagy had a role in SKM after 7d cold-challenge. First, we compared protein expression of autophagy markers (LC3 and p62) and mitochondrial OXPHOS complex protein NDUFB8 (Complex I subunit), SDHB (Complex II subunit), UQCRC2 (Complex III subunit), MTCO2 and COX4 (Complex IV subunits) and ATP5A (Complex V subunit) levels in SKM protein extracts. We did not observe any significant differences in the aforementioned proteins’ expression among SKM under thermoneutrality and cold-challenge, with or without CQ treatment (Supplementary Fig. S3A). Next, we tested mitochondrial dynamic regulating protein OPA1, mitofusin proteins Mfn1 and Mfn2, mitochondrial outer membrane receptor TOM70 and other mitochondrial proteins in SKM mitochondrial extract. These proteins have been reported to be critical to mitochondrial dynamics, i.e., mitochondria fission and fusion and SKM mitochondrial function^21–26^. As shown in Fig. S3B, we observed increased protein level of mitochondrially encoded cytochrome c oxidase 1 (MTCO1) and ATP synthase (ATP5A). We did not observe differences in protein expression of OPA1 (long isoform, membrane bound isoform) among the 4 groups of SKM and we observed slightly decreased OPA1 (short isoform, soluble isoform) in the cold-challenged group (compared to thermoneutrality), in which there are no differences between Vehicle and CQ treatment. Opa1 is critical for the balance between fusion and fission in mitochondrial networks and the observed lower OPA1 short isoform expression in cold-challenged SKM mitochondrial extract suggests cold stimulation might lead to SKM mitochondrial dynamic changes (less mitochondrial fission and more fusion during cold-challenge). MFN2 protein expression is slightly upregulated, which also suggests increased mitochondrial fusion after cold-challenge, with no difference between Vehicle and CQ treated groups. TOM70 expression is not different among the groups. We were not able to detect autophagy markers (LC3 and p62) and mitophagy markers (PINK1 and Parkin) in SKM mitochondrial extracts (data not shown). To measure SKM shivering function, we compared electromyographic activity (indicator of shivering). Similar as previously demonstrated^27^, we observed high muscle activity upon cold-challenge in mouse spinotrapezius muscle (Supplementary Fig. S3E–G). In addition, we observed no shivering activity differences between Veh and CQ treated group after 4 h, 12 h and 7d cold-challenge (Supplementary Fig. S3E–G). EM study did not find significant mitochondrial morphological and ultrastructural changes or mitophagosomes in SKM after 7d cold-challenge (Supplementary Fig. S3C). Most importantly, we did not observe mitochondrial damage in SKM after 7d cold-challenge with CQ treatment (Supplementary Fig. S3C). In addition, we did not observe *map1lc3a* and *sqstm1* mRNA changes in 7d cold-challenged spinotrapezius muscle (Supplementary Fig. S3D). These results show that in contrast to BAT, 7d cold-challenged SKM was activated but demonstrated no changes in mitochondrial ultrastructure, autophagy genes and proteins in the presence or absence of CQ, suggesting a unique role for mitophagy in BAT.

### Enhanced brown adipocyte mitophagy upon β3-agonist stimulation

**Figure 2:**
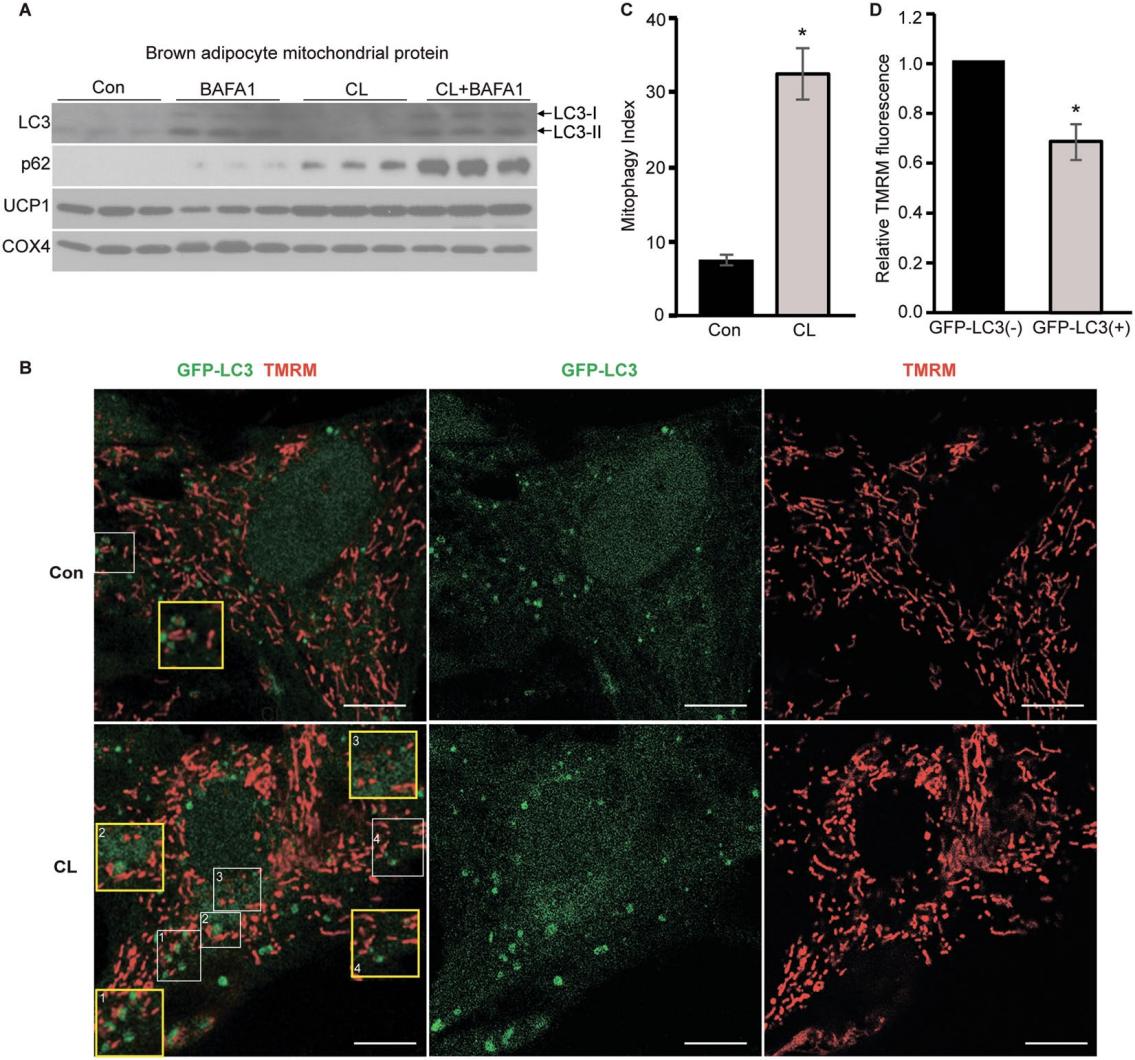
Increased brown adipocyte mitophagy upon stimulation. (**A**) Western blots of LC3 and p62; UCP1 and Cox4 protein levels in cultured BA mitochondrial protein extracts from Con and CL (10uM)-stimulated groups, in the presence and absence of bafilomycin A1 (BAFA1) (n = 3 in each group). (**B**) Representative time lapse confocal fluorescence imaging of BA from GFP-LC3 transgenic mice labelled with TMRM. The cells were imaged in the presence and absence of CL (2.5 uM). During the 4 h experiment, the GFP-LC3 structures encircling TMRM puncta were considered as mitophagy structures (representative mitophagy sturctures are outlined by the white boxes and amplified in the insets in yellow boxes). Scale bars: 5 μm. GFP-LC3 is shown in green and TMRM is shown in red. (**C**) Quantification of mitophagy index in Con and CL-treated BA. Structures of GFP-LC3 encircling TMRM puncta were considered as mitophagy structures and total number of mitophagy structures in each cell during the 4 h live cell imaging were counted and presented as mitophagy index. Experiments were repeated for 3 times. Data are expressed as mean ± SEM as compared to control group. *p < 0.05 by two-tailed Student’s t test. (**D**) Quantification of TMRM fluorescent intensity changes in CL-treated BA. The TMRM intensity of those mitochondria encircled with GFP-LC3 was calculated and normalized to the fluorescence intensity of nearby non-GFP-LC3 co-localized mitochondria. Experiments were repeated for 3 times; Data are expressed as mean ± SEM as compared to the non-GFP-LC3 co-localized (GFP-LC3-) mitochondria in the neighboring region within the same cell. *p < 0.05 by two-tailed Student’s t-test. Con: Control Vehicle; CL: CL316243.

To gain further insight into potential molecular mechanisms, we isolated mice BAT stromal vascular fraction (SVF) cell and differentiated them into mature brown adipocytes (BA) (Supplementary Fig. S4A). We then stimulated mature BA using β_3_-agonist CL316243 (CL) (2.5 μM) for 12 h with or without lysosomotropic reagent bafilomycin A1 (BAFA1) (0.1 µM). β-adrenoceptors are expressed abundantly in BA and the β_3_-adrenoceptor mediated pathway is the most significant in mouse BA activation^2,20,28^. Hence, selective β_3_-adrenoceptor agonist CL is commonly used to mimic cold-challenge *in vitro*^2,20,28^. Similar to our *in vivo* findings, we observed increased LC3 and p62 level in mitochondrial protein extracts upon 12 h CL stimulation. BAFA1 treatment potentiated CL-increased LC3 and p62 level, indicating CL significantly enhanced mitophagy flux in cultured BA (Fig. 2A).

Next, we isolated and differentiated BA from transgenic green fluorescent protein (GFP)-LC3 reporter mice^29^ and investigated mitophagy events in these cells using live cell imaging. LC3 is the most accepted autophagy marker and methods of autophagy visualization are widely used in autophagy studies in which ubiquitously expressed GFP-LC3 forms punctate GFP signal representing autophagosomes^18,29^. In mitophagy studies, when Mito Tracker dyes colocalized with GFP-LC3, it serves as a marker for mitophagy^16,18,29^. This approach allows for monitoring of the kinetics of autophagosome formation, movement and disappearance in living cells^29^. Because loss of mitochondrial membrane potential (*ΔΨm*) is a key feature of mitochondria undergoing mitophagy^30,31^, we co-labelled mitochondria in GFP-LC3 transgenic BA with the *ΔΨm* sensitive fluorescent dye tetramethylrhodamine methyl ester (TMRM; 20 nM) to observe the dynamic of fluorescent signals. TMRM fluorescence revealed heterogeneity in basal *ΔΨm* in different BA, but the fluorescent signal is comparable within the same cell (Supplementary Fig. S4B). To confirm the TMRM sensitivity to BA *ΔΨm*, we initially added 1 μM protonophores carbonylcyanide p-trifluoromethoxyphenylhydrazone (FCCP) to the BA and immediately observed depolarized mitochondria and complete loss of *ΔΨm* (Supplementary Fig. S4C). We next treated the BA with CL (2.5uM) and observed enhanced fluorescent images of GFP-LC3 and TMRM post treatment for 4 h. As compared to the control (Phosphate Buffered Saline, PBS) group, CL treatment significantly increased mitophagy in BA as evidenced by GFP-LC3 encircling the TMRM-labeled mitochondria (mitophagy structure) (Fig. 2B). Mitophagy index, which reflects the total number of mitophagy structures in each cell during the 4 h time span, was significantly increased in CL-treated BA (Fig. 2C). Further, we analyzed the intensity of TMRM florescence in mitophagy structures. To avoid cell-to-cell variability of TMRM fluorescence signal, we normalized the TMRM fluorescence of mitophagy structures (GFP-LC3+) to the non-GFP-LC3 co-localized (GFP-LC3−) mitochondria in the neighboring region within the same cell. As shown in Fig. 2D, the TMRM fluorescence of those mitochondria colocalized with GFP-LC3 undergoing mitophagy was decreased by ~30%. In conclusion, these data suggest mitophagy is enhanced in BA upon β_3_-agonist stimulation to target the mitochondria with dissipated *ΔΨm* for clearance.

### Cold-challenge induction of BAT mitophagy is UCP1-dependent

**Figure 3:**
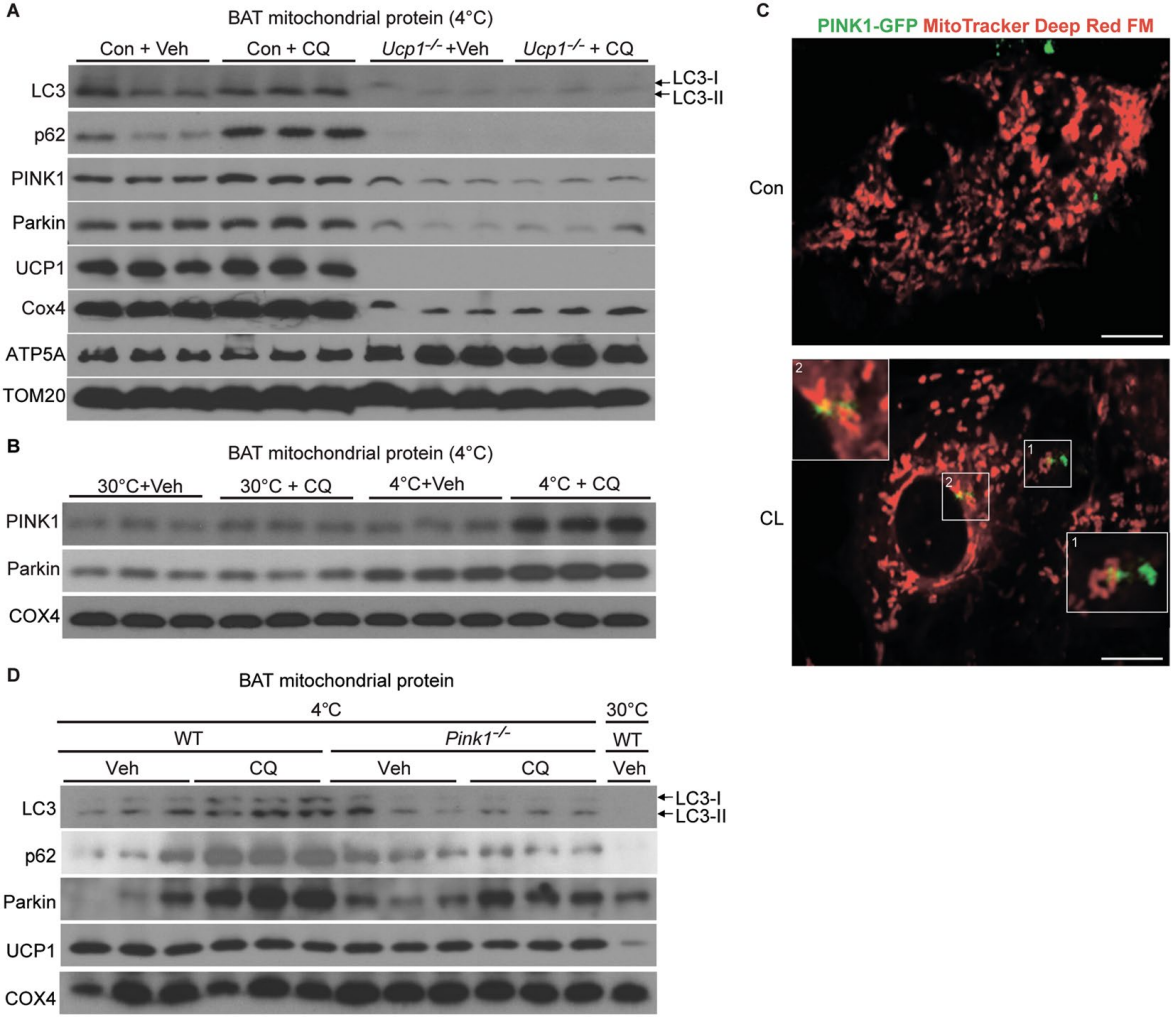
Cold-activated BAT mitophagy is UCP1-dependent and PINK1-mediated. (**A**) Western blots of LC3 and p62; PINK1 and Parkin; UCP1 and Cox4 protein levels in control and *Ucp*^−/−^ mice BAT mitochondrial protein extracts after 7d 4 °C-challenge (n = 3 mice per group). (**B**) Western blots of PINK1 and Parkin levels in 7d CQ-treated and 4 °C-challenged (4 °C + Veh, 4 °C + CQ) and 30 °C-acclimated (30 °C + Veh, 30 °C + CQ) mice mitochondrial protein extracts (n = 3 mice per group). (**C**) Representative image of Con and CL-treated PINK1-GFP-overexpressed BA. Mitochondria are labelled by MitoTracker Deep Red FM. Structures encircling puncta were considered as mitophagy structures. (**D**) Western blots of LC3 and p62; Parkin, UCP1 and Cox4 protein levels in control and *Pink1*^−/−^ mice BAT mitochondrial protein extracts after 7d 4 °C-challenge (n = 3 mice per group) and one sample from 30 °C-acclimated BAT mitochondrial protein extracts as control. Con (3A): control mice; Veh: Vehicle; CQ: Chloroquine; Con (3C): Control Vehicle; CL: CL316243. Scale bars: 5 μm.

Next, we sought to glean insights into the regulation of cold-activated BAT mitophagy. As our studies demonstrated mitophagy in cold-challenged BAT and CL-treated BA (but not at thermoneutrality), we postulated that the observed mitophagy might be linked to mitochondrial inner membrane UCP1-mediated thermogenesis. Since UCP1 is the only protein mediating BA thermogenesis^32^, we challenged the *Ucp1*^−/−^ mice with 4 °C for 7 days to test if cold-challenge induced BAT mitophagy is UCP1-dependent. As shown in Fig. 3A, under cold-challenge, LC3 and p62 expression and autophagic flux in BAT mitochondrial protein extracts were strongly attenuated in *Ucp1*^−/−^ mice, indicating that BAT mitophagy is UCP1-dependent. There are several major differences between control and *UCP1*-null BAT. First, upon BAT activation, UCP1 dissipates the proton motive force to generate heat^32,33^. In contrast, isolated *UCP1*-null BA cannot produce heat^34^ and *UCP1*^−/−^ mice completely lose their heat generation ability through BAT^35^. Heat producing capacity in BA can be approximately two orders of magnitude higher than basal metabolic rate in other mammalian tissues^2^, which can potentially lead to mitochondrial fragmentation and cell damage^36–38^. Second, mitochondrial *ΔΨm* is markedly reduced upon UCP1 activation, which is diminished in mitochondria from *Ucp1*^−/−^ BA^39,40^. It is widely reported that mitochondrial *ΔΨm* dissipation promotes selective mitophagy^41–43^. Third, activated BAT has high oxygen demand during UCP1 activation, leading to BAT hypoxia during cold-challenge^44^. The hypoxia in BAT is significantly hampered in *Ucp1*^−/−^ mice^44^. It is reported that under prolonged hypoxic conditions, mitochondrial autophagy serves as an adaptive metabolic response to prevent cell death^45,46^. Finally, conflicting studies exist regarding the effect of UCP1 on reactive oxygen species (ROS) production^39,47–49^. Therefore, we postulated that during cold-challenge, UCP1 activation produces high thermo-energy, *ΔΨm* reduction and enhanced hypoxia, which might subsequently facilitate mitophagy for damaged mitochondria clearance.

In response to mitochondria damage, mitochondrial stress sensor PTEN-induced putative kinase 1 (PINK1) and its partner E3 ubiquitin ligase (Parkin) are recruited to the mitochondrial membrane to initiate selective mitophagy^50,51^. PINK1 selectively localizes on depolarized mitochondrial membranes through its N-terminal mitochondrial targeting signal (MTS) and promotes Parkin accumulation and the subsequent mitophagy cascade^52–55^. We found that cold-challenged *Ucp1*-null BAT mitochondrial extracts has significantly lower PINK1 and Parkin expression and CQ-induced PINK1 and Parkin accumulation (Fig. 3A). Next, we observed significantly more CQ-potentiated PINK1 and Parkin accumulation in cold-challenged BAT mitochondrial extracts compared to BAT from thermoneutrality, indicating enhanced PINK1/Parkin mediated mitophagic flux (Fig. 3B). Further, we infected BA using PINK1-GFP virus and labelled mitochondria with MitoTracker Deep Red FM. We observed punctate PINK1-GFP accumulated around mitochondria after CL-stimulation, indicating enhanced mitophagy in activated BA (Fig. 3C). Collectively, these results indicate that cold-induced BAT mitophagy is facilitated by UCP1, and possibly mediated by mitochondrial stress sensor PINK1 and Parkin.

As a next step, we investigated the importance of PINK1-mediated mitophagy in cold-challenged BAT using *Pink1*^−/−^ mice. Under thermoneutrality, compare to *Wild Type* (*WT*) mice, *Pink1*^−/−^ mice did not show differences in BAT and SKM mitochondrial ultrastructure (Supplementary Fig. S5B), the expression of autophagic markers LC3, p62 in the presence or absence of CQ treatment (Supplementary Fig. S5C) and the core body temperature (Supplementary Fig. S5D). We then subjected *WT* and *Pink1*^−/−^ mice to cold-challenge for 7 days in the presence or absence of CQ treatment. We observed significantly less mitophagic flux (LC3, p62, and Parkin expression) in cold-challenged *Pink1-null* mice BAT mitochondrial protein extracts after CQ treatment (Fig. 3D). In conclusion, these data suggest that enhanced mitophagy in cold-challenged BAT is mediated by mitochondrial UCP-1 activation and may be dependent on the mitochondrial stress sensor PINK1. It has been reported in other adipose depots, i.e., beige adipose, mitochondrial stress sensor Parkin were differentially regulated in responded to CL stimulation^56^. The role of UCP-1 in beige fat mitophagy is unknown and will be a subject of our future studies.

### Cold-activated BAT mitophagy is coupled to mitochondria biogenesis and is critical for activated BAT integrity and brown adipocyte function

**Figure 4:**
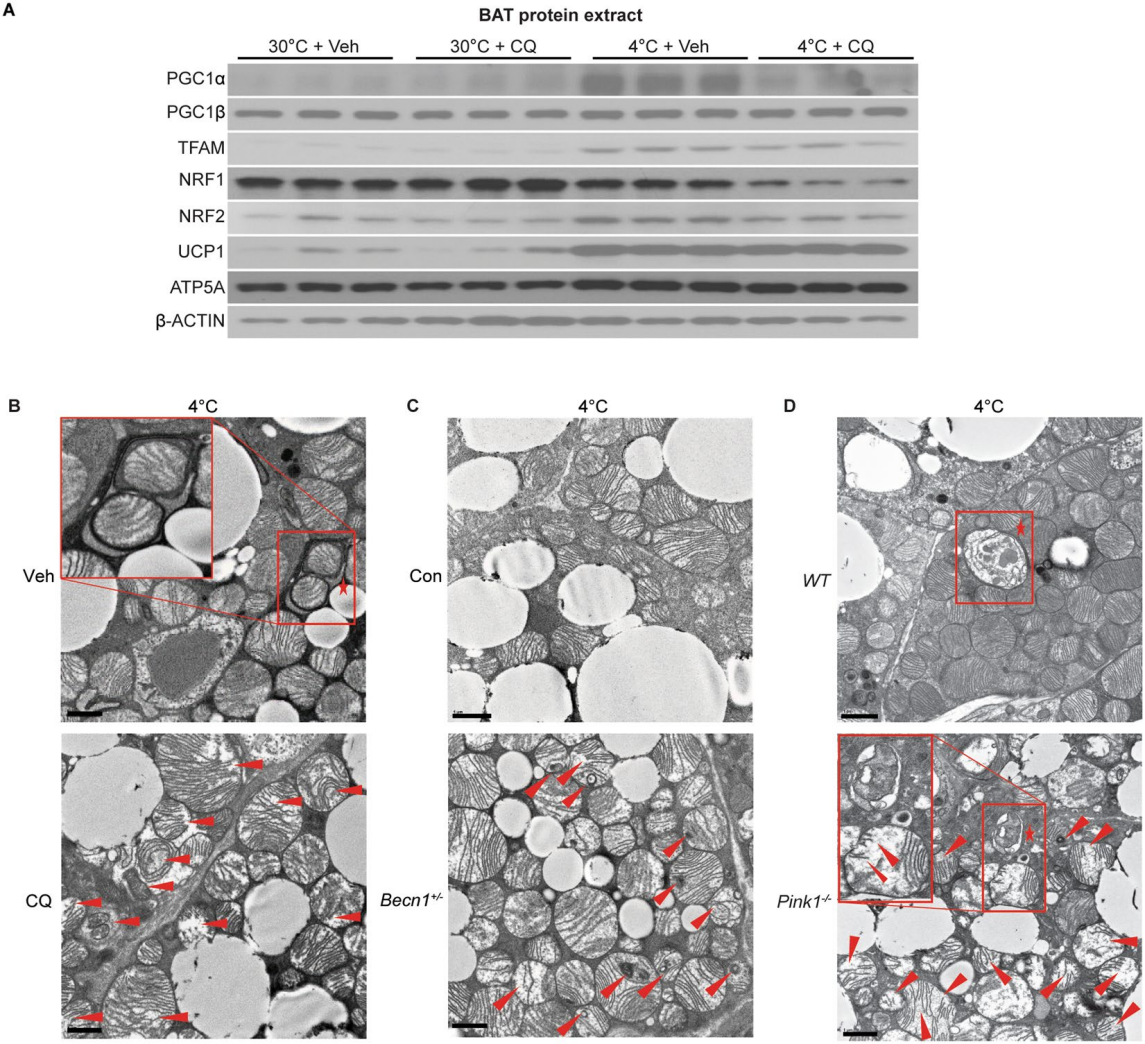
Cold-activated BAT mitophagy coordinates with mitochondrial biogenesis and is critical for BAT mitochondrial homeostasis. (**A**) Western blots of key transcriptional activators for BAT mitochondrial biogenesis program PGC1α, PGC1β, TFAM, NRF1 and NRF2; mitochondrial proteins ATP5A, UCP1 and β-actin in BAT protein lysate from 7d 30 °C-acclimated (30 °C), 4 °C-challenged (4 °C), with or without CQ-treatment (30 °C + CQ and 4 °C + CQ) (n = 3 mice in each group). (**C**) Representative EM pictures of Veh or CQ-treated BAT mitochondria after 7d 4 °C-challenge (n = 3 mice per group). (**D**) Representative EM pictures of *Becn*^*+/−*^ and control mice (n = 3 mice per group) BAT mitochondria after 7d 4 °C-challenge. (**E**) Representative EM pictures of *Pink1*^−/−^ and WT mice (n = 3 mice per group) BAT mitochondria after 7d 4 °C-challenge. Mitophagosomes are marked by star, outlined by the red box and amplified in the inset. Damaged mitochondria are indicated by the red arrows. CQ: chloroquine; Veh: vehicle. Scale bars: 1 μm.

Our data suggest that BA mitophagy occurs in response to UCP-1-mediated mitochondrial stress. Previous studies indicate that the maintenance of a healthy mitochondria population requires both degradation of damaged mitochondria as well as the generation of new replacement (i.e., mitochondria biogenesis)^14,57^. Enhanced BA mitochondrial biogenesis during cold-challenge is well documented^14^. While traditionally investigated separately, recent evidence suggests that the two processes of mitochondrial biogenesis and degradation are coordinated^57^. Accordingly, we hypothesized that mitophagy and mitochondrial biogenesis are coordinated to maintain mitochondrial homeostasis in cold-challenged BAT. Examination of key regulators of the mitochondrial biogenic program revealed that mRNA and protein expression levels of peroxisome proliferator-activated receptor gamma coactivator 1 (PGC1) alpha, mitochondrial transcription factor A (TFAM), and nuclear respiratory factor 2 (NRF2)^20,58^ were significantly elevated after 7d cold-challenge, but repressed by CQ treatment (Fig. 4A and Supplemental Fig. S6). Nuclear respiratory factor 1 (NRF1) mRNA expression level was increased but protein level did not increase accordingly after 7d cold-challenge, which might be due to posttranslational regulation. NRF1 protein level was also inhibited by CQ after cold-challenge. PGC1β mRNA expression was increased by 1.5 fold after cold-challenge but protein level was not affected by cold-challenge and/or CQ treatment (Fig. 4A and Supplemental Fig. S6). In addition, we observed significantly enhanced UCP1 expression and modestly increased ATP5A in whole cell protein extracts after cold-challenge (Fig. 4A).

Next, we investigated if mitochondrial function is impacted by mitophagy inhibition. We pretreated mature BA with BAFA1 (0.1 µM) or vehicle for 1 h and then stimulated with CL (1 µM) for 4 h. We then tested BA mitochondrial OX-PHOS capacity by measuring oxygen consumption rate (OCR). Compared to the control group, BAFA1 treated BA OCR capacity decreased by ~20% after CL stimulation (Supplementary Fig. S7A) suggesting that mitophagy plays a significant role in maintaining proper BA mitochondria function after CL activation.

To determine the *in vivo* significance of BAT mitophagy, we undertook pharmacologic and genetic approaches to block autophagy and assess for effects on mitochondrial ultrastructure and core body temperature. First, *C57BL6/J* mice were exposed to 4 °C for 7 days in the presence or absence of CQ (30 mg/kg/day). We observed significant amount of damaged mitochondria in CQ-treated and cold-challenged mice BAT, i.e., mitochondria with cristae membrane configuration changes, damaged cristae membrane and accumulation of electron-dense materials (Fig. 4B). Coincident with BAT mitochondria ultra-structure changes, the core body temperature in CQ-treated mice was lower by 0.5 °C after 7d cold-challenge (Supplementary Fig. S7B). We then assessed mitochondrial ultrastructure and core body temperature in a genetic autophagy deficient mouse model–*Becn1*^+/−^ mice^59^. 10-week-old *Becn1*^+/−^ and littermate control mice were cold-challenged for 7 days. Significant mitochondrial ultrastructure damages were observed (Fig. 4C) and accompanied by a core body temperature decrease of 0.5 °C in *Becn1*^+/−^ mice after cold-challenge (Supplementary Fig. S7C). By contrast, *Becn1*^+/−^ mice exposed to 30 °C demonstrated no effect on core body temperature or mitochondrial ultrastructure (Supplementary Fig. S7D and F). Lastly, our EM study in cold-challenged *Pink1*-null BAT also detected an accumulation of abnormal mitochondria that were fragmented, swollen and mitochondria with abnormal cristae coexisting with mitophagosome (Fig. 4D). Consistent with our previous observations, *Map1lc3a* mRNA expression was upregulated in both *WT* and *Pink1*^−/−^ BAT after cold-challenge, and it was slightly higher in *Pink1*^−/−^ BAT vs. in *WT* (Supplementary Fig. S7G). *Sqstm1* mRNA expression level was higher at thermoneutrality but exhibited no difference in *Pink1-null* BAT compare to *WT* BAT after cold-challenge (Supplementary Fig. S7G). *Ucp1* mRNA expression was upregulated in both groups upon cold-challenge (Supplementary Fig. S7G). These results indicate that decreased mitophagic flux in *Pink1*^−/−^ BAT is not regulated at the transcriptional level. The core body temperature and SKM mitochondrial ultrastructure were not different from *WT* and *Pink1*^−/−^ mice upon 7 day cold-challenge (Supplementary Fig. S7E and S7H). In conclusion, using three different autophagic/mitophagic mechanism deficient mouse models, we observed insufficient mitophagy led to destruction of mitochondrial integrity in activated BAT, indicating mitophagy is critical for BAT mitochondrial population health maintenance during cold-challenge.

It is interesting that we still observed some mitophagosome in cold-challenged *Pink1*-null BAT (Fig. 4D, outlined in red box and marked by star), which indicates there are other mitochondrial receptors function redundantly to mediate mitophagy in activated BAT. It has been reported that other mitochondrial receptors such as voltage-dependent anion channel proteins (VDACs), Mfn2, Miro, and PHB2 serve as E3 ubiquitin ligase Parkin receptors mediating mitophagy^60–63^. Also, other E3 ubiquitin ligase, i.e., SMURF1 also mediate mitophagy and SMURF1-deficient mice heart, brain and liver accumulate damaged mitochondria^64^. In addition, some other receptors might work independently of ubiquitination in a cell-specific fashion, e.g., the BCL2-related protein Nix and FUNDC1 are mitochondrial receptors which directly interact with LC3 mediating mitophagy^65–67^. Nix is required for reticulocytes mitochondrial clearance for red blood cell differentiation and maturation. Although more than half of the reticulocytes contain mitochondria and undegraded autophagosomes in the *Nix*^−/−^ mice, the other half were featureless like *WT* erythrocytes and ultimately clear their mitochondria, indicating the exist of redundant mechanisms in reticulocytes mitopahgy^65,66^.

Collectively, in the present study, we report for the first time that enhanced mitophagy is requisite for eliminating UCP1-activation mediated mitochondrial damage in activated BAT. Further, we show that Pink1-deficiency leads to insufficient mitophagic flux in activated BAT. BAT functional studies indicate that inhibition of mitophagy decreases activated BA mitochondrial OX-PHOS capacity. In addition, our data suggest that mitophagy coordinates with mitochondrial biogenesis to maintain mitochondrial turnover and homeostasis to ensure BAT mitochondria population health in cold-activated BAT.

## Materials and Methods

### Animal Studies

All animal studies were performed with the approval from Case Western Reserve University Institutional Animal Care and Use Committee (IACUC). All methods were performed in accordance with the relevant guidelines and regulations. 12–14 week-old *C57BL/6* *J* mice were from Jackson laboratory as used for the cold-challenged if not otherwise mentioned. The *Ucp1* knock-out mice (*B6*.*129-Ucp*^*tm1Kz*^*/J*) mice were obtained from Jackson laboratory (genetic background 129S1/SvlmJ; Stock No. 002488; F1) and backcrossed one time to C57BL/6 J to generate *UCP1*^*+/−*^ (F2). All of the experimental UCP1KO mice and their litter mate control wild type mice (F3) were inbred from UCP1^+/−^ (F2) mice. The *Becn1*^+/−^ (*B6*.*129* × *1-Becn1*^*tm1Blev*^*/J*) mice were from Jackson laboratory and backcrossed to *C57BL/6 J*. F2 generation from the breeding colony were used for the experiment and littermate controls were used as control animals for the same experiments. The GFP-LC3 mice were from Riken Japan (RBRC00806)^68^ and backcrossed to *C57BL/6 J*. F3–5 generation from the breeding colony were used for the cell isolation. The *PINK1*^−/−^ (*B6*.*129S4-Pink11tm1shn/J*) mice were from Jackson laboratory and inbred for experiments. *C57BL/6 J* mice were used as control mice as suggested by the Jackson laboratory. Mice were housed in a temperature- and humidity-controlled specific pathogen–free facility with a 12-hour-light/dark cycle and ad libitum access to water and standard laboratory rodent chow. All the experimental mice were randomized into different experimental groups and were kept at 30 °C prior cold-challenge before transferred to 4 °C for one week. We observed less 10% of the mice were cold sensitive and lost their body temperature rapidly after cold-challenge. We eliminated the cold-sensitive mouse from our study when their body temperature dropped below 30 °C. Gene expression, protein expression analysis and temperature measurements were carried out on 12–14 week-old male mice. Cold-exposure experiments were performed in IACUC-approved cold room set at 4 °C; warm-exposure experiment were performed in Wessels induction/warming chamber set at 30 °C; both with a 12 h light/dark cycle and ad libitum access to water and standard laboratory rodent chow. For the Chloroquine (CQ) (Alfa Aesar J64459) treatment experiments, mice were injected CQ 30 mg/kg/day subcutaneously once per day for 7 days. Male mice were used for the experiments.

### Temperature Measurement

Core body temperature of mice were obtained using surgically implanted telemetric transmitters from Data Sciences International (DSI, New Brighton, MN, USA). Sterilized DSI wireless telemetric transmitter TA-F10 was implanted into the abdominal cavity after mice were anesthetized by isoflurane. Following a week of convalescence, mice were acclimated to the designated temperature and temperature measurements were recorded. Excessive activity/movement were observed during resting-phase (6am-6pm) in 4 °C-challenged group compared to 30 °C-acclimated group, but the activity during active-phase (6am-6pm) in 4 °C-challenged group is comparable to 30 °C-acclimated group. Hence core body temperatures at mice active-phase (6pm-6am) were averaged and used as comparison.

### Electromyogram Measurement

Electromyogram of mice were obtained using surgically implanted telemetric transmitters from Data Sciences International (DSI). Sterilized DSI wireless telemetric transmitter ETA-F10 was implanted into mice anesthetized by isoflurane. 2–3 mm exposed stainless-steel biopotential leads were inserted into the spinotrapezius muscle; two leads were 3–4 mm apart and the insulated EMG wire sutured to the adjacent muscle to prevent wire movement. Following a week of convalescence, mice were acclimated to the designated temperature and EMG, activity and temperature measurements were recorded. EMG signals were noise-filtered, root mean square (RMS) was analyzed with NeuroScore software (DSI).

### Histology and Electronic Microscopic Studies

Tissue samples were fixed in 10% neutralized formalin and embedded with paraffin following standard protocols. H&E staining is performed following standard protocol. Electronic microscopic samples were fixed in triple aldehyde-DMSO for 2 h at room temperature. Samples were postfixed in ferrocyanide-reduced osmium tetroxide for 2 h at room temperature after rinsing in 0.1 M phosphate buffer (pH 7.3). Samples were then soaked for 12 h at 4 °C in acidified uranyl acetate following a water rinse. After rinsing again in distilled water, the tissue blocks were dehydrated in ascending concentrations of ethanol, passed through propylene oxide, and embedded in Poly/Bed resin (Polysciences). Sections were sequentially stained with acidified uranyl acetate followed by a modification of Sato’s triple lead stain^69,70^. The sections were examined in a FEI Tecnai Spirit (T12) transmission electron microscope with a Gatan US4000 4k × 4k CCD. All EM images were analyzed by blinded observers from Electron Microscopy Facility, Case Western Reserve University.

### Western blot

Interscapular and subscapular BAT tissues were dissected and white adipose tissue was carefully trimmed off under the microscope. Tissues were rinsed using PBS twice. Total cell lysate protein from tissues was extracted using RIPA buffer (Sigma-Aldrich, R0278) and mitochondrial protein was isolated using Thermo Scientific Mitochondria Isolation Kit for tissues or cells (ThermoFisher 89801 and 89874). Specifically, 800 μL Mitochondria isolation reagent A was added to 100–200 mg BAT tissue or 10^7^ fully differentiated BA cells. Dounce homogenization was performed on ice for 50–100 strokes and the suspension was transferred to an Eppendorf tube. 800 μl Mitochondria isolation reagent C was added and the tube was inverted several times and then centrifuged at 700 × g for10 min at 4 °C. The supernatant was transferred to a new Eppendorf tube and centrifuged at 12,000 × g for 15 min. Supernatant fraction was transferred to a separated tube (cytosolic proteins) and mitochondrial pellet was washed using wash buffer (50% isolation reagent C and 50% PBS) twice, lysed with 2%CHAPS in 150 mM NaCl and 25 mM Tris buffer (pH 7.2). Protease inhibitors and phosphatases inhibitors (Roche, 4693132001 and 4906845001) were added to all the buffers. Protein concentration was measured by BCA protein assay kit. 10–20ug of protein was loaded for WB. The following antibodies were used: ATP5a (Abcam, ab14748), COX IV (Cell Signaling, 4844), LC3B (Cell Signaling, 3868), PINK1 (Santa Cruz, sc-32584), Parkin (Santa Cruz, sc-32584), SQSTM1/p62 (Cell Signaling, 5114), TOM20 (Santa Cruz, sc-17764), UCP1 (Abcam, ab14748), PGC1α (EMD Millipore ST1202), PGC1β (Abcam, ab176328), TFAM(Sigma ABE4837), NRF1 (Abcam, ab175932), NRF2 (Abcam, ab80845), Total OXPHOS Rodent Antibody for NDUFB8, SDHB, UQCRC2 (Abcam, ab110413), OPA1 (Abcam, ab42364), MFN1 (Abcam, ab126575), MFN2 (Santa Cruz, sc-50331), Tom70 (Novusbio, 87863), α-Tubulin (Sigma T9026), HRP-linked anti-rabbit IgG (Cell Signaling, 7074), and HRP-linked anti-mouse IgG (Cell Signaling, 7076). UCP-1, COX4, ATP5A antibodies were diluted to 1:5000. All of the other primary antibodies were diluted to 1:1000. Secondary antibody were diluted to 1:2000-1:4000. The purity of mitochondrial protein extraction was confirmed by western blot before further analysis. There was no cross contamination from cytosolic protein to mitochondrial protein. All the experimental results were repeated at least for 3 times with independent group of samples.

### Adenovirus Construction

pcDNA-DEST47 PINK1C-GFP plasmid donated by the Cookson Lab^71^ were obtained from Addgene, pAAV-Pink1-GFP adenovirus was construction by Welgen Inc. Briefly, pcDNA-DEST47 PINK1C-GFP and pAAV-CMV-GFP plasmid was amplified, digested and ligated. The positive clones were screened and sequenced. Virus was produced from monolayer of 293 cells by calcium phosphate co-transfection method using pAAV-Pink1-GFP, pHelper and pAAV8-RC vectors. The purified AAV was used for experiments.

### Cell culture and cell-based assay

Preadipocytes of brown adipose tissue were collected from brown fat depots of 3–4 week old mice. Depots were minced finely and incubated with 0.2% collagenase in 37 °C shaking water bath for 30–45 min. Cells were purified through 40 μm strainer (Falcon), stromal vascular fraction cells (SVF) were pelleted and re-suspended in Growth Medium (DMEM + 15%FBS) (Hyclone). BA differentiation was induced upon confluence with differentiation medium (Growth medium supplemented with 50 nM insulin, 5 nM T3 and 1 μM rosiglitazone (SigmaAldrich)). After 5d’s differentiation, cells were thoroughly rinsed and cultured in DMEM + 15%FBS for experiment. Cell mitochondrial respiration rate in differentiated brown adipocytes were assessed using a Seahorse XFp Extracellular Flux Analyzer with the XFp Cell Mito Stress Test Kit (Seahorse Bioscience-Agilent). Oxygen consumption rate (pmol/min) was measured and balanced to the cell count per well (pmol/min/1000cells). BAFA1 treated groups are compared to Vehicle treated groups. Four independent experimental results were averaged.

### Live imaging of mitophagy in BA

Stromal vascular fraction cells were isolated from *WT* or *LC3-GFP* mice suprascapular BAT tissue and plated on gelatin (0.01% gelatin) coated glass bottom dishes (MatTek Inc.) in growth media (DMEM + 15%FBS) (Hyclone). After 1 day, media was replaced with aforementioned differentiation medium and thereafter media was changed with differentiation media every 24 h. Experiments were carried on fully differentiated cells between 4–6 days after differentiation. For *LC3-GFP* cell, on the day of experiment, BAT cells were washed with Tyrode’s buffer (TB) 3 times and loaded with mitochondrial membrane potential sensitive fluorescence dye Tetramethylrhodamine methyl ester (TMRM; 20 nM) for 45 min in TB at 37 °C. For BA Pink1-GFP overexpression, 2 × 10^10^ GC/Well PAAV-Pink1-GFP virus were added to differentiated BA (Day4) for additional 72 h. On the day of experiment, BA were washed with TB 3 times and were loaded with 200 nM Mitotracker Deep Red FM dye (Thermofisher) for 30 min. The cells were then washed three times with TB. To avoid photo-bleaching, laser power was kept at 1% for 488 nm (Pink1-GFP or GFP-LC3) and 2% for 647 nm (Mitotracker Deep Red FM) or 568 nm (TMRM) channels respectively. Cells were imaged for mitophagy using confocal microscope (Nikon A1R) at 63xOil objective lenses with 1.4NA. A field containing 2–3 cells was selected and treated with vehicle (PBS) or CL (2.5 uM) and time lapse imaging was performed for by capturing 1image/min for 4 hrs. TMRM fluorescence from those mitophagy regions of interest (ROIs) was quantified using NIH image J software. TMRM fluorescence from neighboring regions of mitophagy structures served as internal control. All experiments were repeated for at least 3 times in independently cultured cells.

### RNA extraction and qPCR

Tissue samples were homogenized in TRIzol reagent (Life Technologies, 15596-026) with a TissueLyser (Qiagen). Total RNA was extracted, treated with DNase I (Life Technologies, 18068015), and reverse transcribed to complementary DNA using the M-MuLV Reverse Transcription Kit (NEB). DNA is extracted by using DNeasy Blood and Tissue kit (Qiagen 69504). qPCR was performed with the TaqMan method (Roche Universal ProbeLibrary System) on a ViiA 7 Real-Time PCR System (Applied Biosystems). Relative expression was calculated using the ΔΔCt method with normalization to β-actin. All the experiments were repeated by at least 3 times by using independent samples. Autophagy PCR array was from Qiagen (PAMM-084Z) and beta-actin was used as control gene. The data were analyzed by GeneGlobe Data Analysis Center (Qiagen). RNA samples were from 3 cold-challenged mice and 4 mice from thermoneutrality.

### Study approval

All animal studies were approved by the Institutional Animal Care and Use Committee of Case Western Reserve University. All methods were performed in accordance with the relevant guidelines and regulations.

### Statistics

Results are presented as Mean ± SEM unless otherwise indicated. Two-tail Student’ *t* test was used to compare the differences between two groups. One-way ANOVA analysis was used for multiple comparisons. Statistical significance was defined as p < 0.05.

## Electronic supplementary material

Supplemental Information

Supplemental upcropped Western Blots
