# Supplementary material for: Mitophagy is required for brown adipose tissue mitochondrial homeostasis during cold challenge
Source: Sci Rep. 2018 May 29;8:8251. doi: 10.1038/s41598-018-26394-5 (PMC5974273; doi:10.1038/s41598-018-26394-5)
Supplement: Supplementary file 1 — Supplemental Information [file 41598_2018_26394_MOESM1_ESM.pdf]

Mitophagy is required for brown adipose tissue mitochondrial homeostasis during cold challenge

Yuan Lu[1], Hisashi Fujioka[2], Dinesh Joshi[3], Qiaoyuan Li[4], Panjamaporn Sangwung[1], Paishiun Hsieh[1], Jiyun Zhu[5], Jose Torio[1], David Sweet[1], Lan Wang[6], Shing Yan Chiu[3], Colleen Croniger[6], Xudong Liao[1], Mukesh K. Jain[1]

1 Cardiovascular Research Institute, Department of Medicine, Case Western Reserve University School of Medicine and Harrington Heart and Vascular Institute, University Hospitals Cleveland Medical Center, Cleveland, Ohio, USA

2 Electron Microscopy Facility, Case Western Reserve University, Cleveland, Ohio, USA

3 Department of Neuroscience, University of Wisconsin School of Medicine and Public Health, Madison, Wisconsin, USA

4Department of Cardiology, Beijing Anzhen Hospital, Beijing Capital Medical University, Beijing, China

5 Illinois Mathematics and Science Academy, Aurora, IL, USA

6 Department of Nutrition, Case Western Reserve University School of Medicine, Cleveland, Ohio, USA.

Address correspondence to: Yuan Lu and Mukesh K. Jain, Cardiovascular Research Institute, Case Western Reserve University School of Medicine, 2103 Cornell Rd, Cleveland, OH, 44106, USA. Phone: 216.368.4774 (YL) and 216.368.2036 (MKJ); E-Mail: yuan.lu@case.edu (YL) and mukesh.jain2@case.edu (MKJ).

Supplemental Material

Supplemental Figure S1.

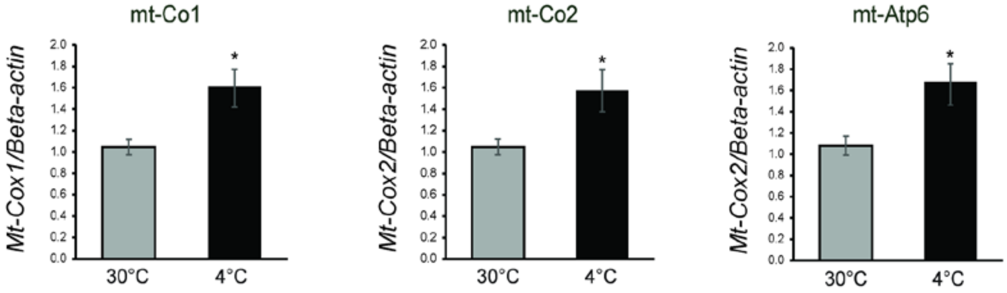

Supplemental Figure S1. Increased mitochondrially encoded DNA markers in cold-challenged BAT. Mitochondrially encoded gene mt-CO1, mt-CO2 and mt-ATP6 levels from 7d 4°C-challenged or 30°C-acclimated BAT. n = 4 mice per group. Data are expressed as mean ± SEM. * $p<0.05$ by two-tailed Student's t-test as compared to BAT DNA extracts from thermoneutrality.

Supplemental Figure S2.

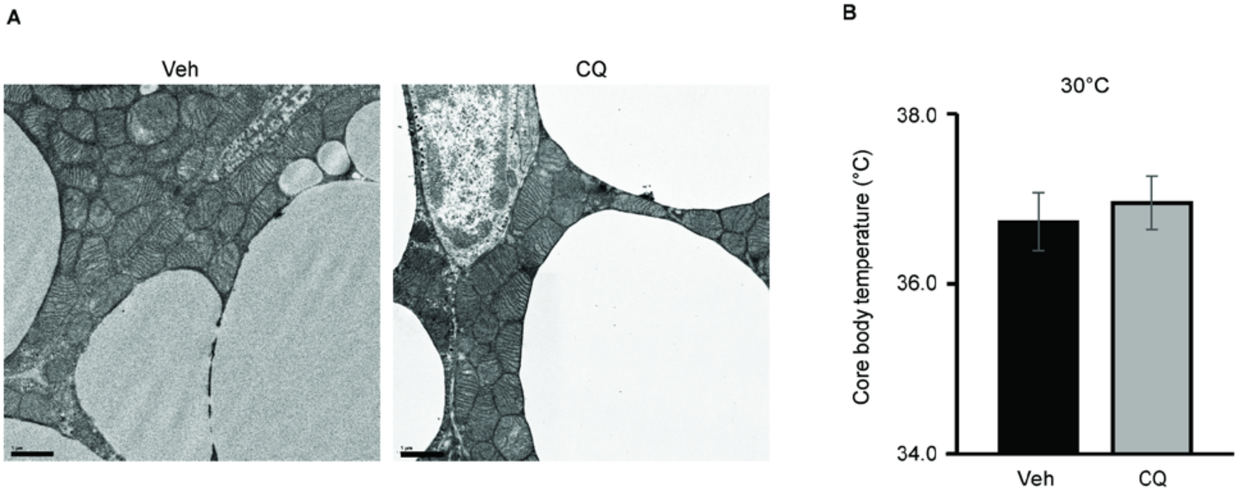

Supplemental Figure S2. Chloroquine treatment does not change BAT mitochondrial ultrastructure and core body temperature at thermoneutrality. A. Representative BAT EM pictures from 30°C-acclimated, Veh (0.9% Saline) or CQ-treated mice (n=3 mice per group). **B.** Mice core body temperature in 30°C-acclimated, Veh (0.9% Saline) or CQ-treated mice (n=3 mice per group). Data are expressed as mean ± SEM. Student's t-test was used for statistical analysis as compared to Vehicle-treated group. Scale bars: 1μm. Veh: vehicle; CQ: chloroquine.

Supplemental Figure S3.

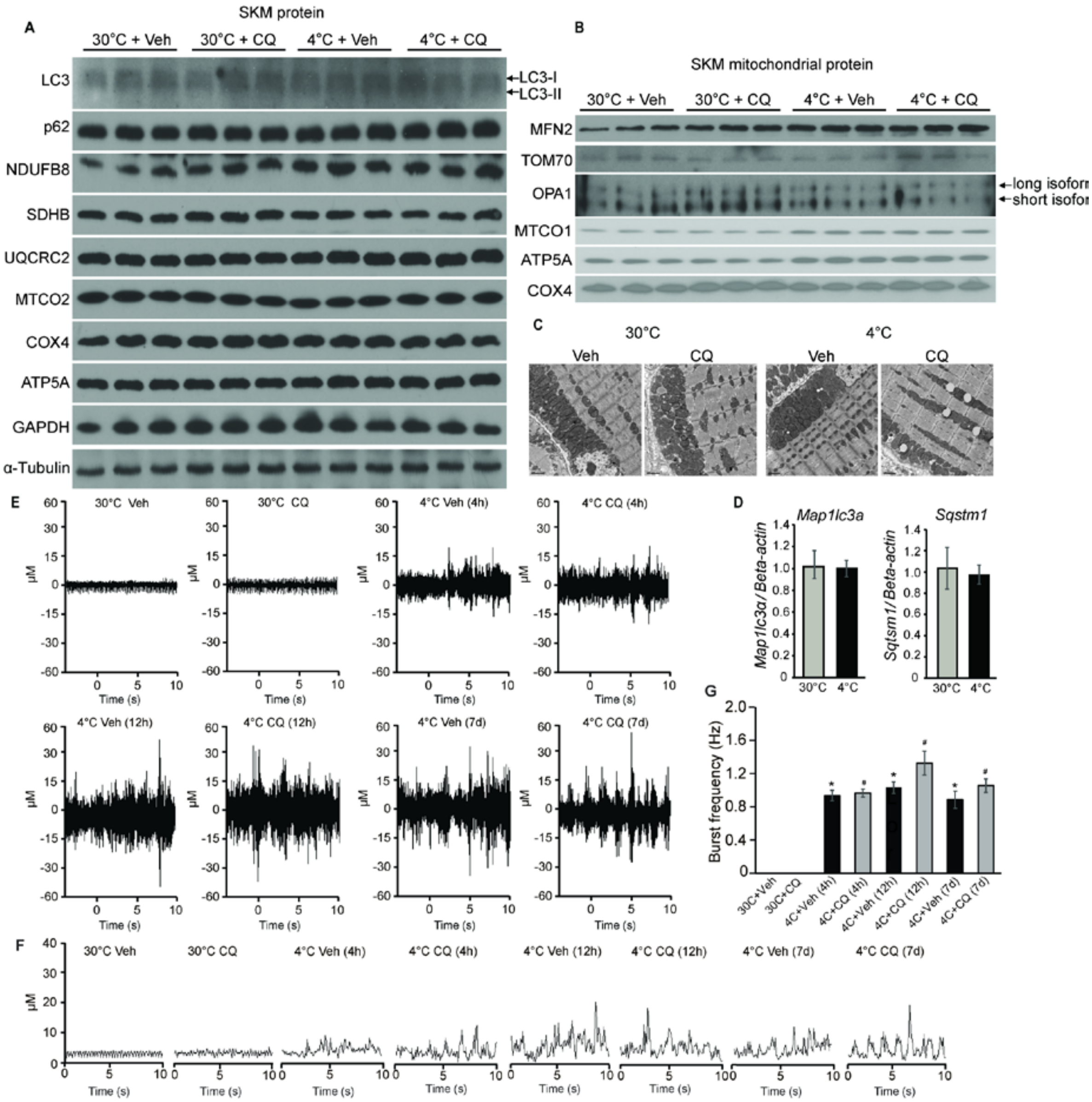

Supplemental Figure S3. Measurement of skeletal muscle function during cold-challenge. A. Western blot of SKM protein extracts (n=3 mice in each group). **B.** Western blot of SKM mitochondrial protein extracts (n=3 mice in each group). **C.** Representative EM pictures of SKM mitochondria from 7d 4°C-challenged or 30°C-acclimated mice, with Veh (0.9% Saline) or CQ treatment (n=3 mice per group). Scale bars: 1μm. **D.** SKM *Map1lc3a* and *Sqstm1* mRNA levels (n=5 mice per group). Data are expressed as mean ± SEM. Student's t-test was used for statistical analysis as compared to 30°C-acclimated group. **E&F.** Representative mouse SKM raw (E) and Root Mean square (F) EMG under thermoneutrality or cold-challenge with Veh or CQ treatment (n=3 mice per group). **G.** Quantification of muscle burst frequency determined by SKM EMG under different conditions (n=3 mice per group). Data are expressed as mean ± SEM. One-way ANOVA analysis was used for multiple comparisons. *$p<0.05$ compared to 30°C Veh-treated group; # $p<0.05$ compared to 30°C CQ-treated group. SKM: skeletal muscle; Veh: vehicle; CQ: chloroquine; EMG: electroymyography.

Supplemental Figure S4.

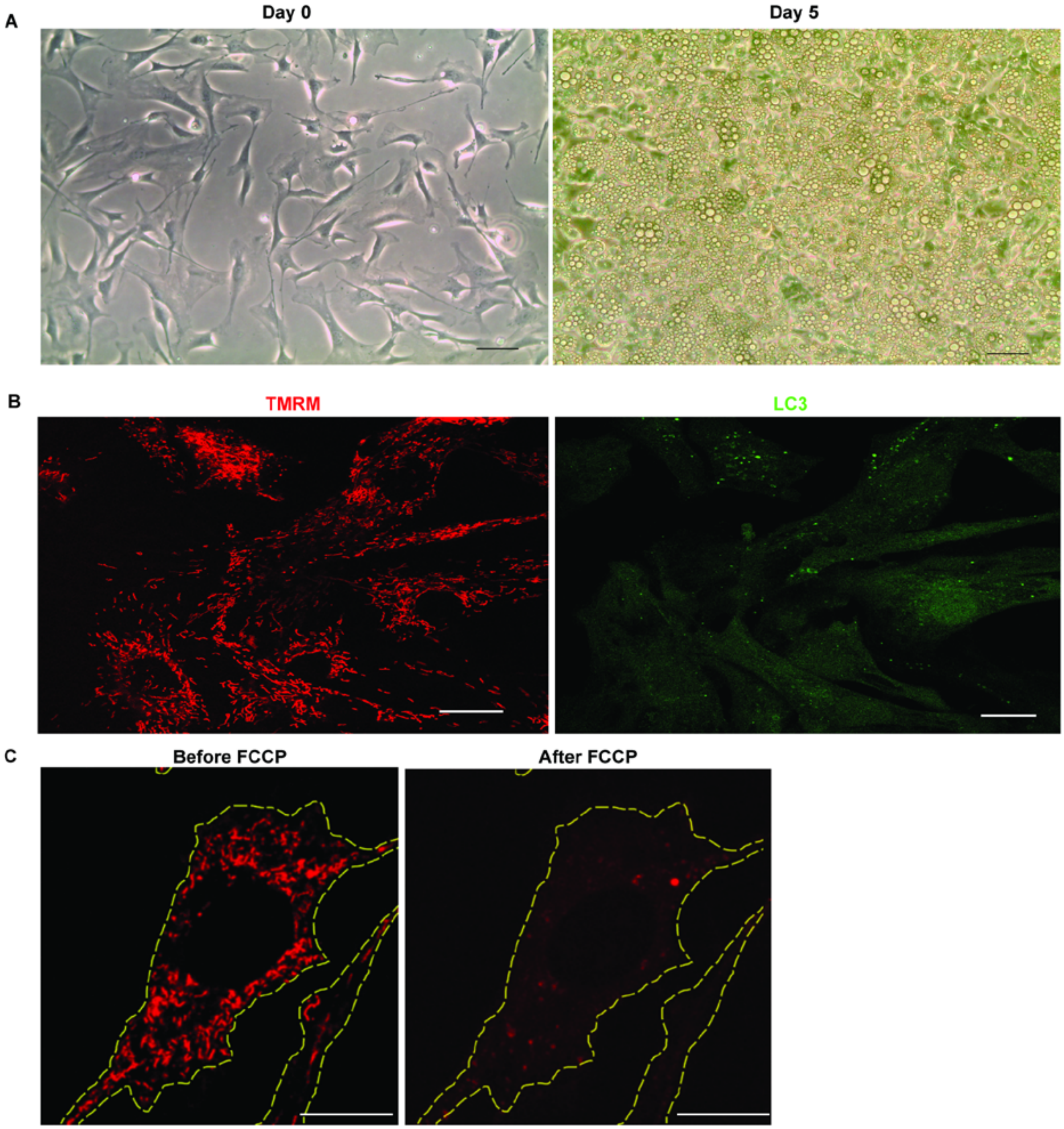

Supplemental Figure S4. Differentiated brown adipocytes and mitochondrial labelling.
A. Isolated BAT stromal vascular fraction (SVF) cells (left) were differentiated into brown adipocytes (right) after 5 days. **B.** Differentiated GFP-LC3 brown adipocytes were labelled with TMRM (20nM) (left). **C.** 1μM protonophores carbonylcyanide p-trifluoromethoxyphenylhydrazone (FCCP) immediately depolarized brown adipocyte mitochondria and led to complete loss of TMRM fluorescence (right). Scale bars: A: 40μm; B: 5μm; C:10μm.

Supplemental Figure S5.

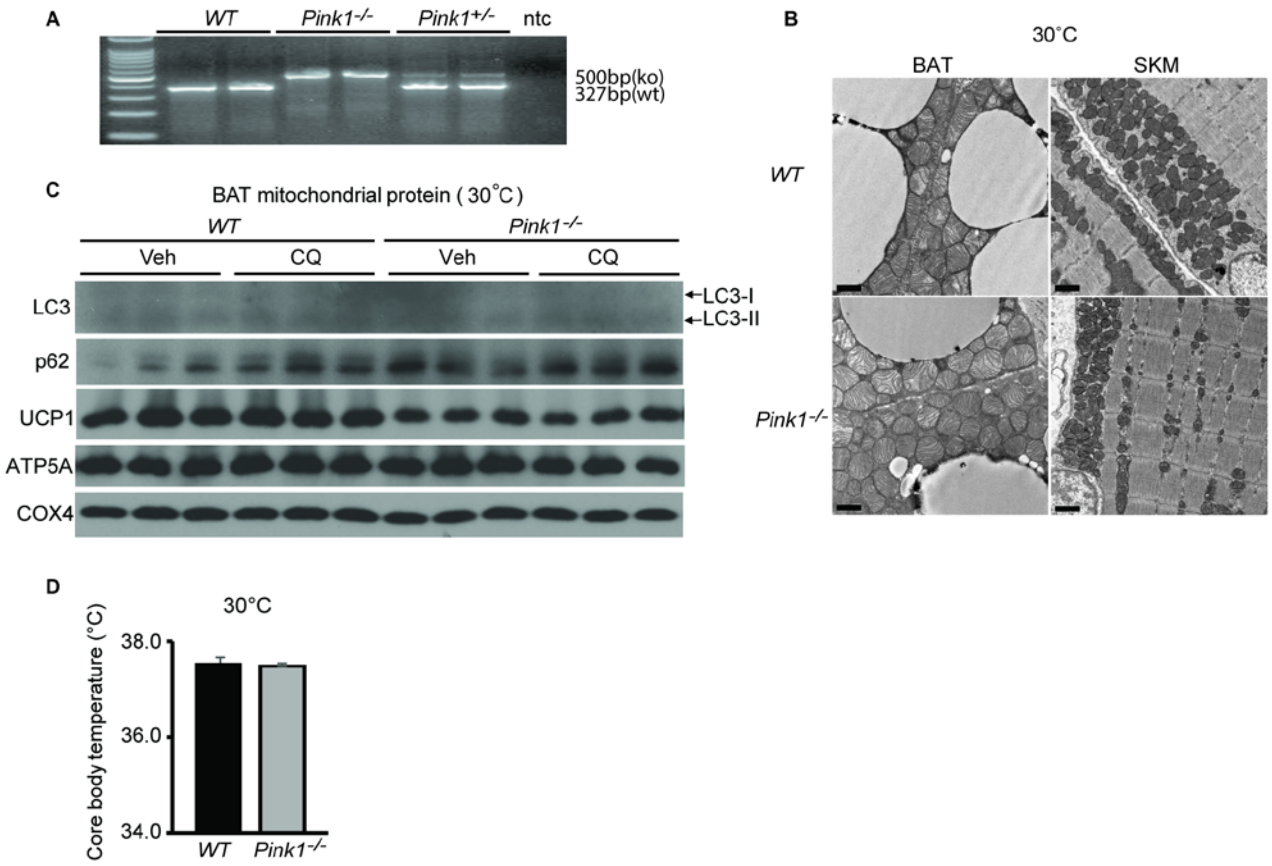

Supplemental Figure S5. Parenmeters in *Pink1*$^{-/-}$ mice. A. Representative genotyping PCR of *Pink1*$^{-/-}$ mouse line. **B.** EM pictures of *WT* or *Pink1*$^{-/-}$ BAT and skeletal muscle at thermonetrality (n=3 mice in each group). Scale bar: 1μm. **C.** Western blots of BAT mitochondrial protein extracts from 7d Veh or CQ-treated mice at thermoneutrality (n=3 mice in each group). **D.** Mice core body temperature from *WT* or *Pink1*$^{-/-}$ mice under thermoneutrality (n=3 mice in each group). Data are expressed as mean ± SEM. Student's t-test was used for statistical analysis as compared to *WT* group. Veh: Vehicle; CQ: Chloroquine.

Supplemental Figure S6.

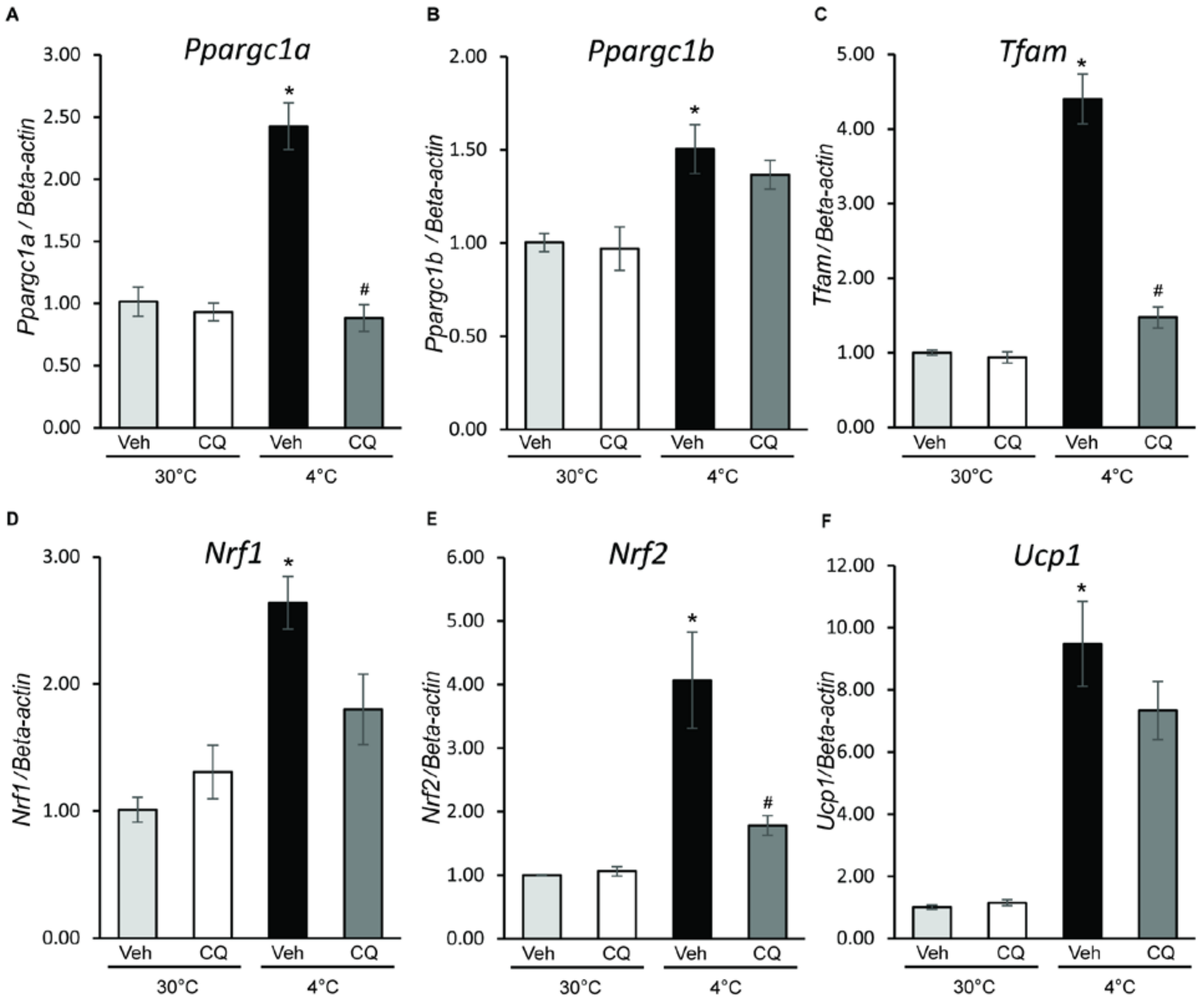

Supplemental Figure S6. Mitochondrial biogenesis regulating genes expression after cold -challenge and chloroquine treatment. A. *Ppargc1a*; **B.** *Ppargc1b*; **C.** *Tfam*; **D.** *Nrf1*; **E.** *Nrf2* and **F.** *Ucp1* mRNA levels under different conditions. Data are expressed as mean ± SEM. n=3 mice per group. * $p<0.05$ by two-tailed Student's t-test as compared to 30°C-acclimated and Veh-treated group; # $p<0.05$ by two-tailed Student's t-test as compared to 4°C-challenged and Veh-treated group. Veh: Vehicle; CQ: chloroquine.

Supplemental Figure S7.

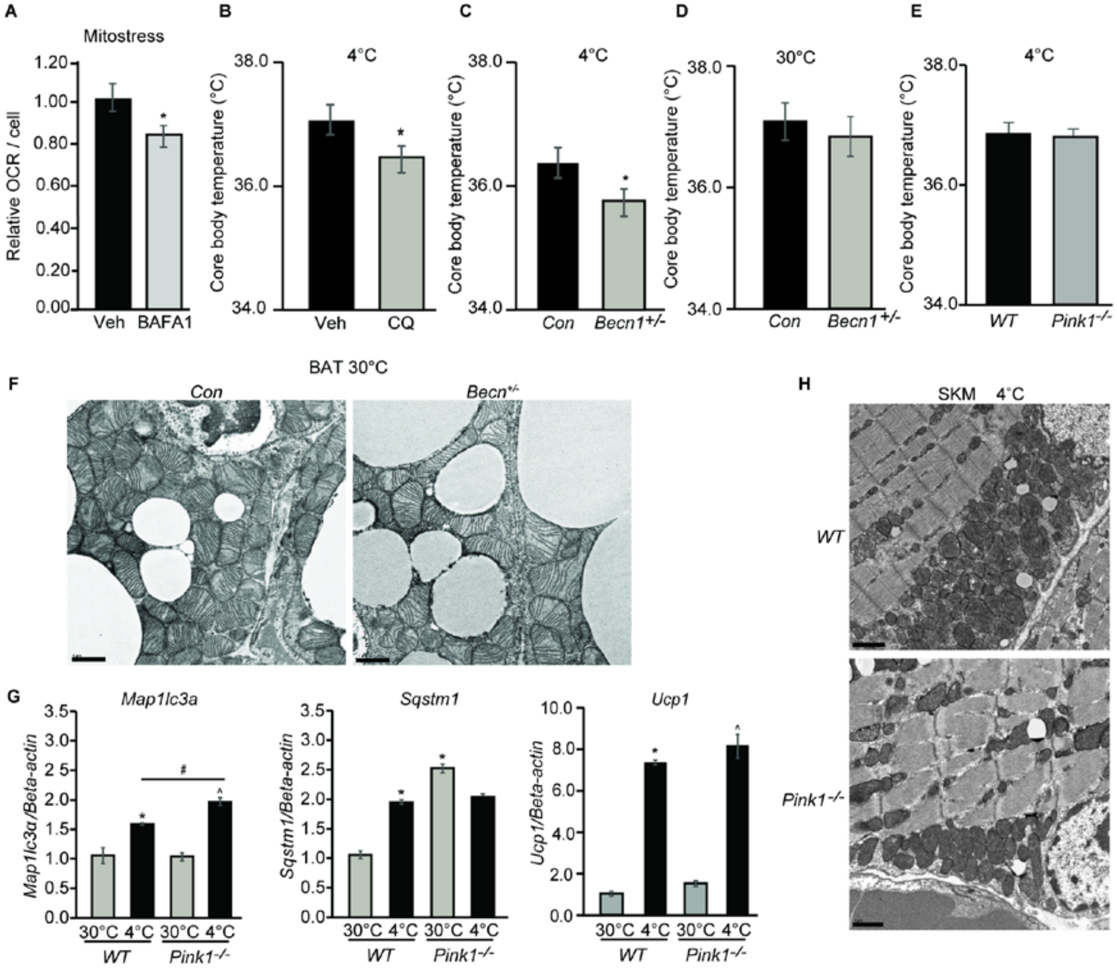

Supplemental Figure S7. Effect of autophagy or mitophagy inhibition in BAT and skeletal muscle. **A.** Decreased mitochondrial oxygen consumption rate (OCR) in BAFA1 (0.1µM) and CL-treated (1µM; 4h) brown adipocytes (n=4 individual experiments). Data are expressed as mean ± SEM. * $p<0.05$ by two-tailed Student's t-test as compared to Veh group. **B.** Mice core body temperature from 7d 4°C-challenged; Veh or CQ-treated mice (n=3 mice in each group). Data are expressed as mean ± SEM. *$p<0.05$ by two-tailed Student's t-test as compared to Veh-treated group. **C&D.** Mice core body temperature from 7d 4°C-challenged (C) or 30°C-acclimated (D); *Con* or *Becn*$^{+/-}$ mice (n=3 mice in each group). Data are expressed as mean ± SEM. *$p<0.05$ by two-tailed Student's t-test as compared to *Con* group. **E.** Mice core body temperature from 7d 4°C-challenged; *WT* or *Pink1*$^{-/-}$ mice (n=3 mice in each group). Data are expressed as mean ± SEM. Student's t-test were used for statistical analysis as compared to *WT* group. **F.** Representative BAT EM pictures from *Con* or *Becn*$^{+/-}$ mice under thermoneutrality (n=3 mice per group). **G.** BAT *Map1lc3a*, *Sqstm1* and *Ucp1* mRNA levels under thermoneutrality or 7d 4°C-challenge. n=3 mice per group. Data are expressed as mean ± SEM. Two-tailed Student's t-test were used for statistic analysis. * $p<0.05$ as compared to *WT* BAT under thermoneutrality; ^ $p<0.05$ as compared to *Pink1-null* BAT under thermoneutrality; # $p<0.05$ as compared to 7d 4°C-challenged *WT* BAT. **H.** Representative SKM EM pictures of *WT* and *Pink1*$^{-/-}$ mice after 7d 4°C-challenge (n=3 mice per group). Scale bars:1µm. Veh: Vehicle; BAFA1: bafilomycin A1; CQ: chloroquine; Con: Control; *WT*: wild type.
