# Supplementary material for: Mitophagy is required for brown adipose tissue mitochondrial homeostasis during cold challenge
Source: Sci Rep. 2018 May 29;8:8251. doi: 10.1038/s41598-018-26394-5 (PMC5974273; doi:10.1038/s41598-018-26394-5)

Mitophagy is required for brown adipose tissue mitochondrial homeostasis during cold challenge

Yuan Lu[1], Hisashi Fujioka[2], Dinesh Joshi[3], Qiaoyuan Li[4], Panjamaporn Sangwung[1], Paishiun Hsieh[1], Jiyun Zhu[5], Jose Torio[1], David Sweet[1], Lan Wang[6], Shing Yan Chiu[3], Colleen Croniger[6], Xudong Liao[1], Mukesh K. Jain[1]

1 Cardiovascular Research Institute, Department of Medicine, Case Western Reserve University School of Medicine and Harrington Heart and Vascular Institute, University Hospitals Cleveland Medical Center, Cleveland, Ohio, USA

2 Electron Microscopy Facility, Case Western Reserve University, Cleveland, Ohio, USA

3 Department of Neuroscience, University of Wisconsin School of Medicine and Public Health, Madison, Wisconsin, USA

4Department of Cardiology, Beijing Anzhen Hospital, Beijing Capital Medical University, Beijing, China

5 Illinois Mathematics and Science Academy, Aurora, IL, USA

6 Department of Nutrition, Case Western Reserve University School of Medicine, Cleveland, Ohio, USA.

Address correspondence to: Yuan Lu and Mukesh K. Jain, Cardiovascular Research Institute, Case Western Reserve University School of Medicine, 2103 Cornell Rd, Cleveland, OH, 44106, USA. Phone: 216.368.4774 (YL) and 216.368.2036 (MKJ); E-Mail: yuan.lu@case.edu (YL) and mukesh.jain2@case.edu (MKJ).

Supplementary data for uncropped Western Blot

Fig 1D:

LC3

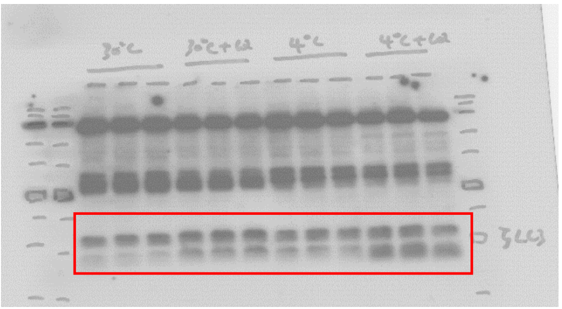

UCP1

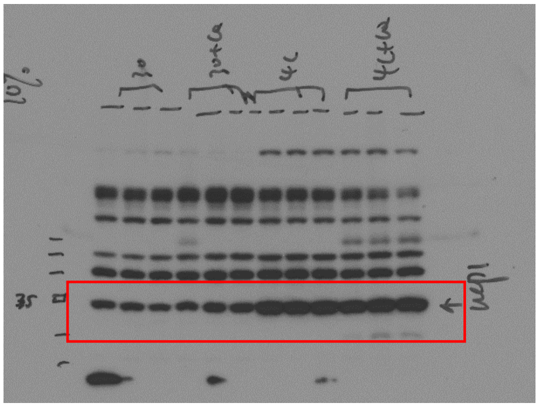

COX4

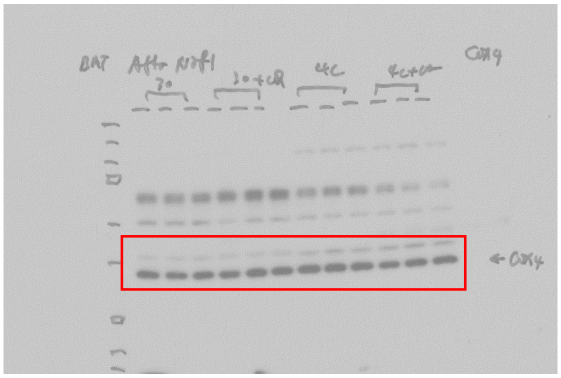

αTubulin

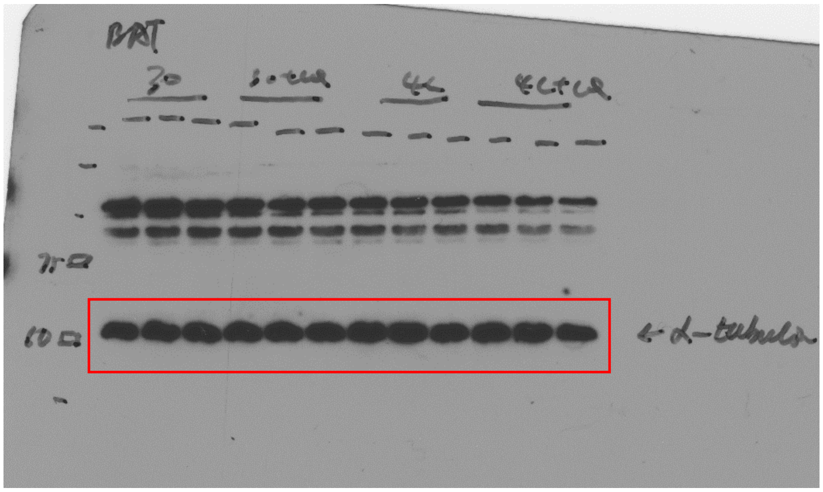

LC3

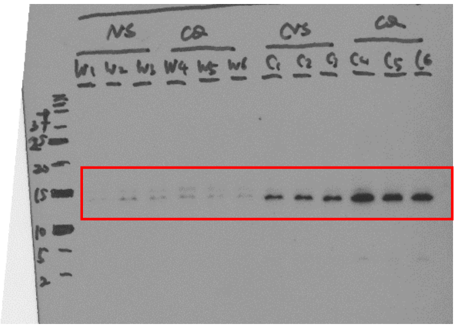

p62

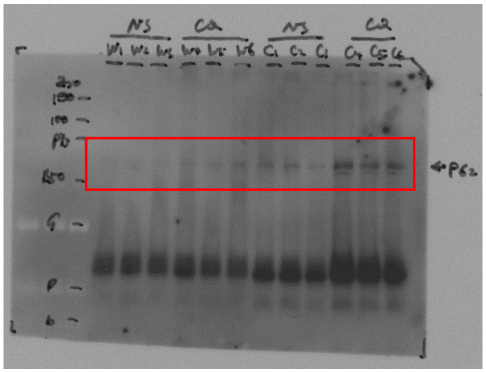

UCP1

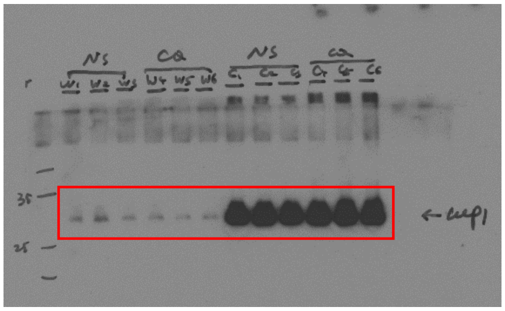

COX4

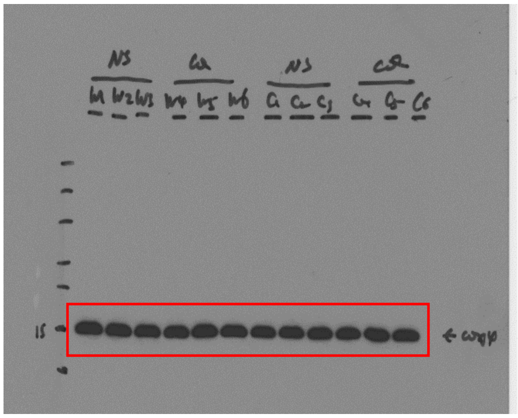

Fig2A.

LC3

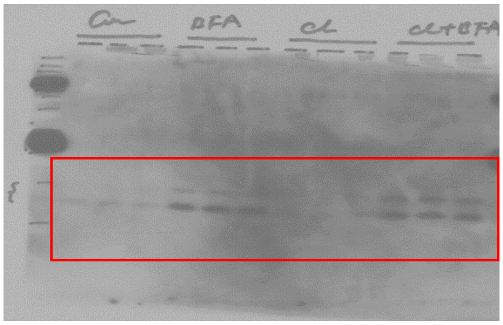

p62

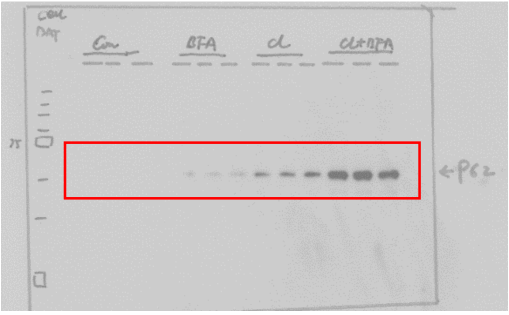

UCP1

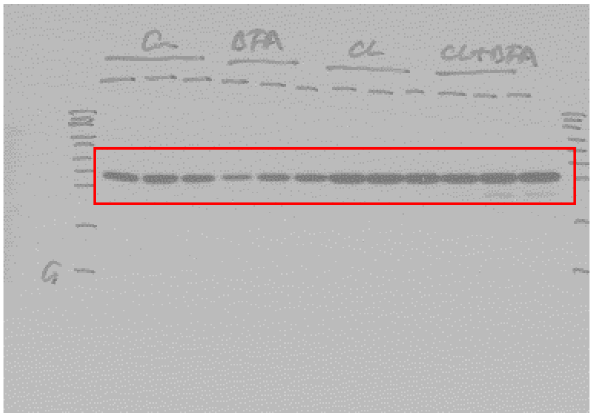

COX4

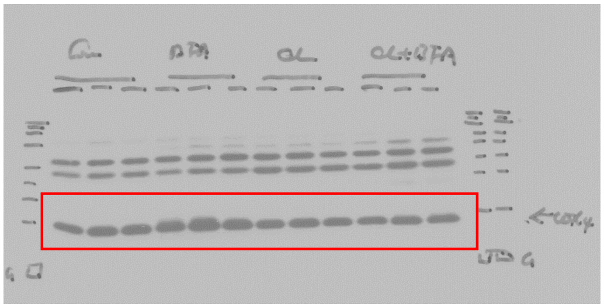

Fig 3A:

LC3

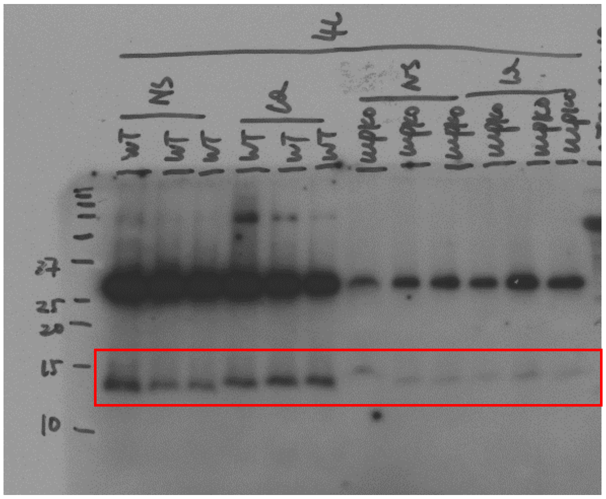

p62

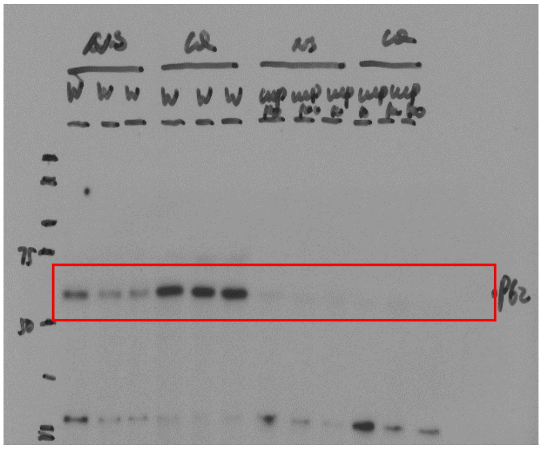

Pink1

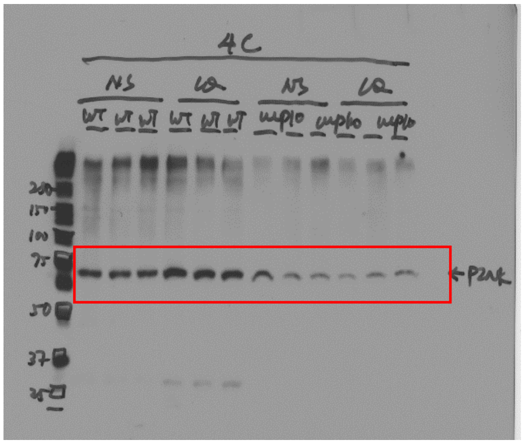

Parkin

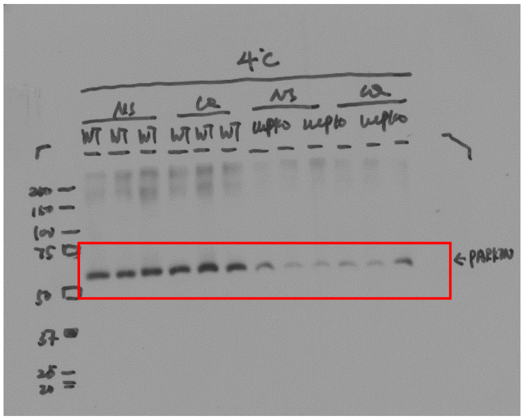

UCP1

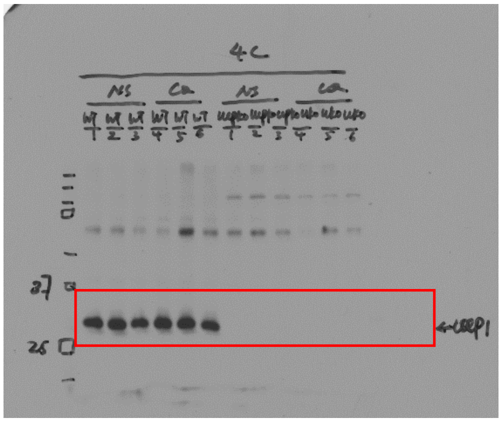

COX4

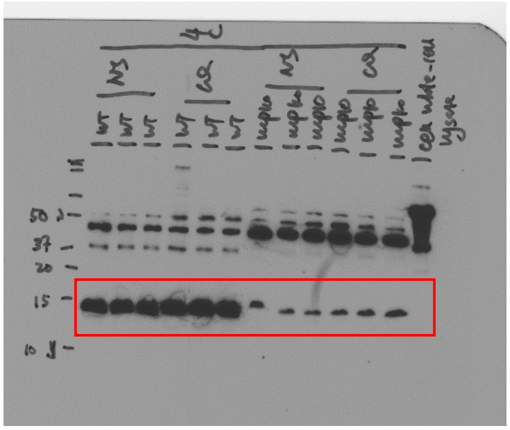

ATP5A

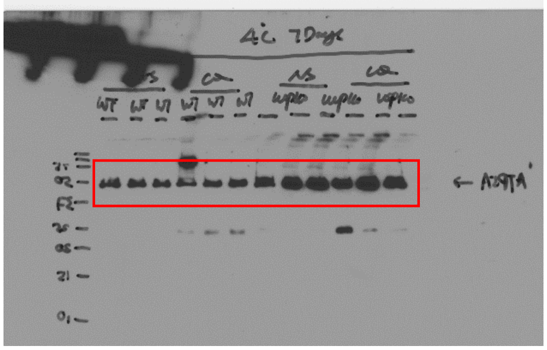

TOM20

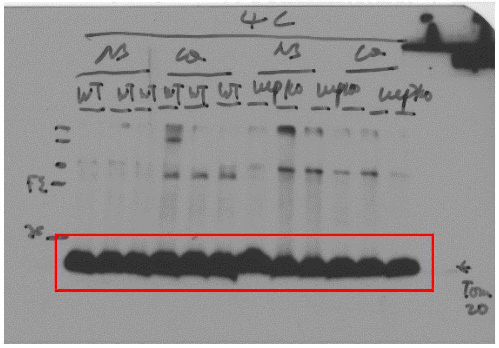

Fig 3B

PINK1

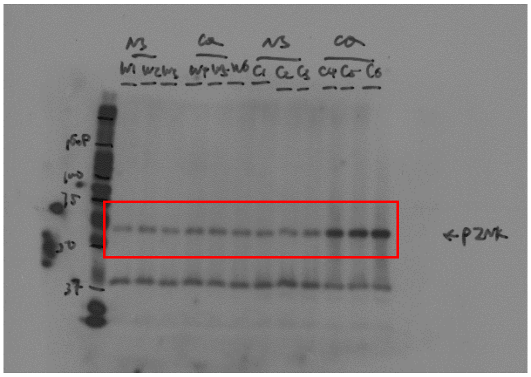

PARKIN

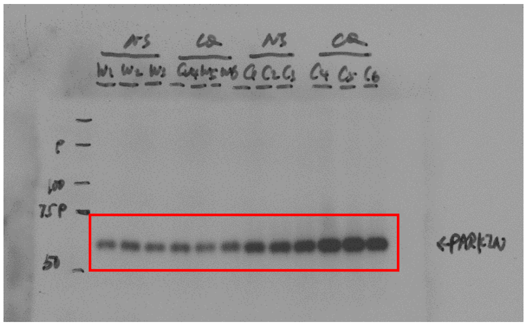

COX4 (Same as Fig 1D COX4, same sample set)

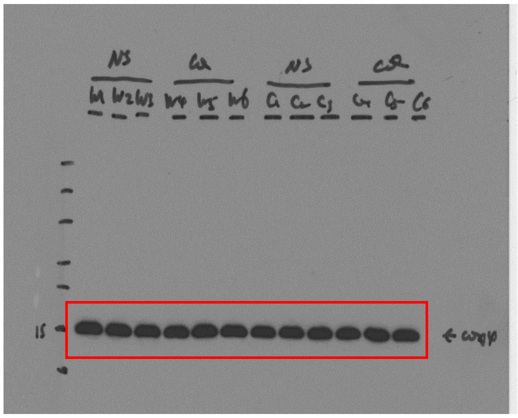

Fig 3D

LC3

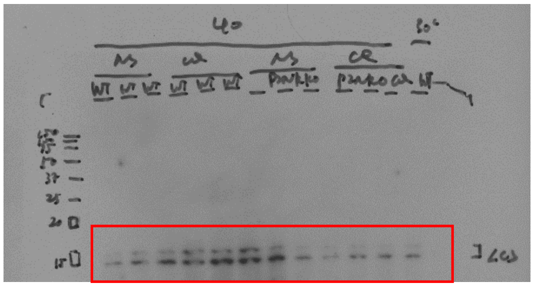

p62

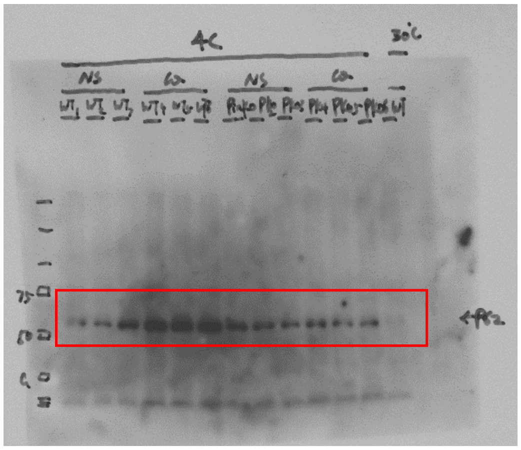

PARKIN

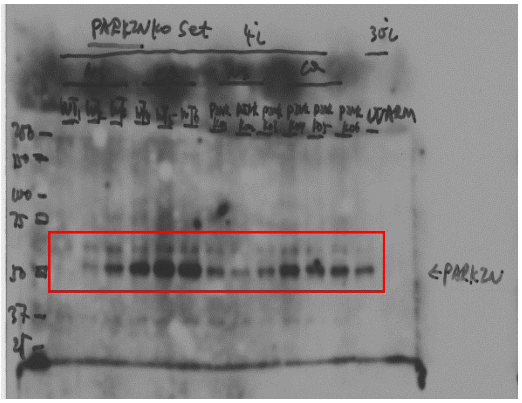

UCP1

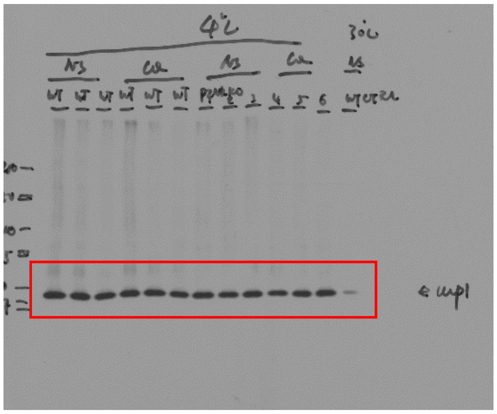

COX4

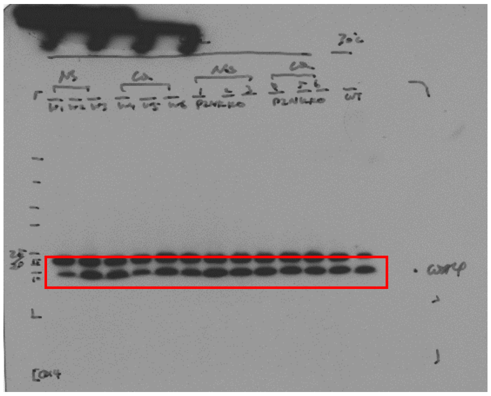

Fig 4A.

PGC1a

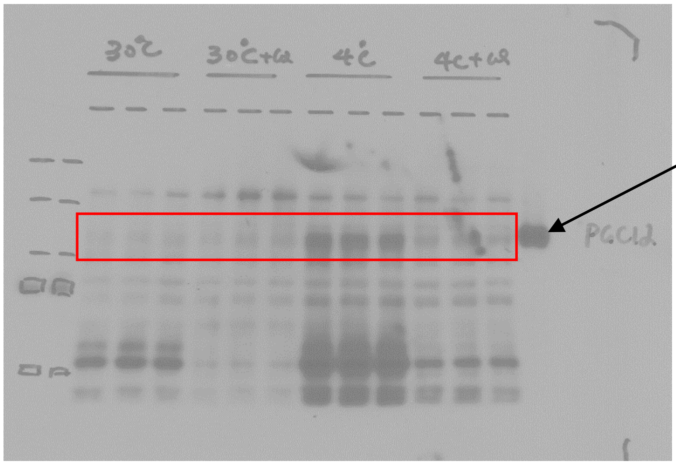

This is a PGC1a control protein provided by company.

PGC1b

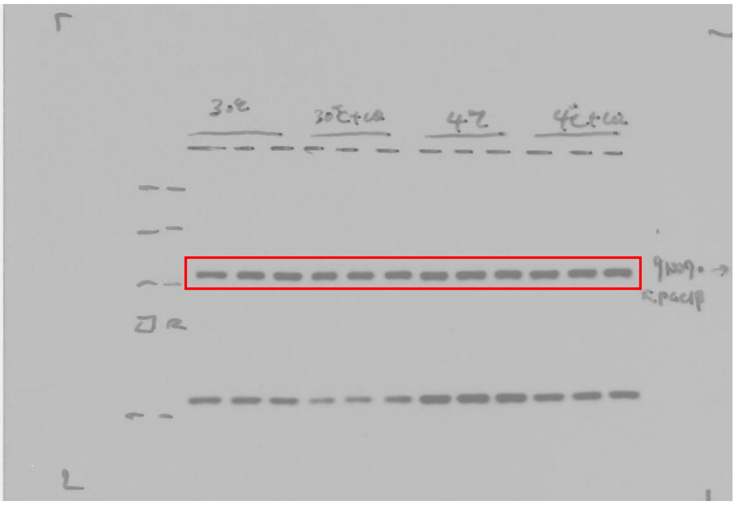

TFAM

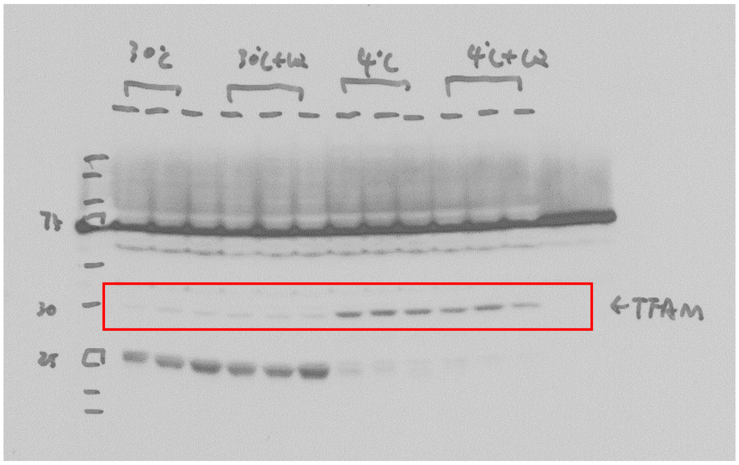

NRF1

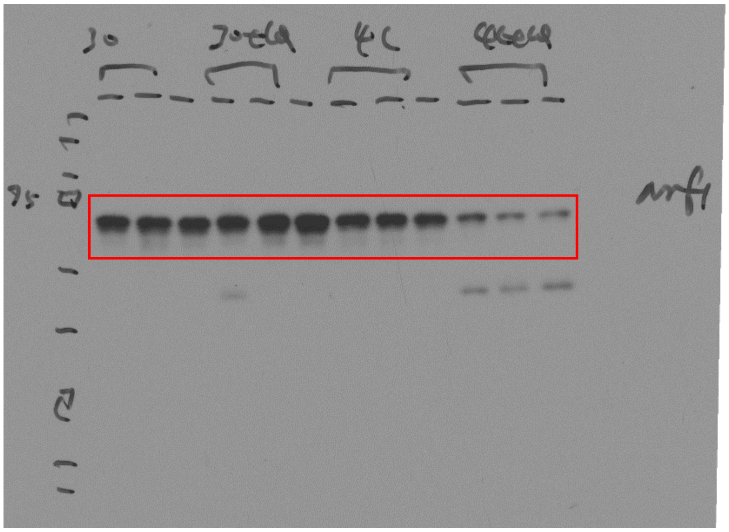

NRF2

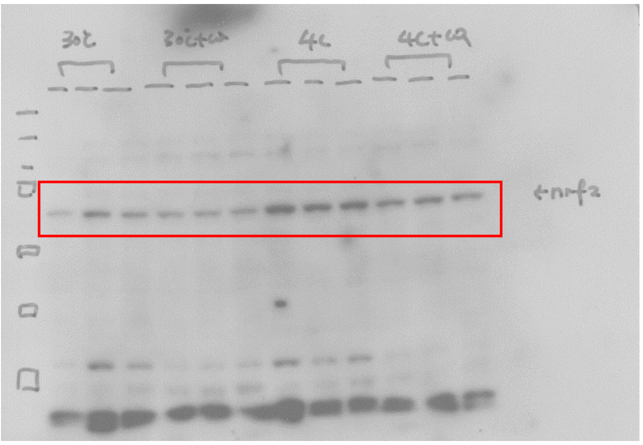

UCP1

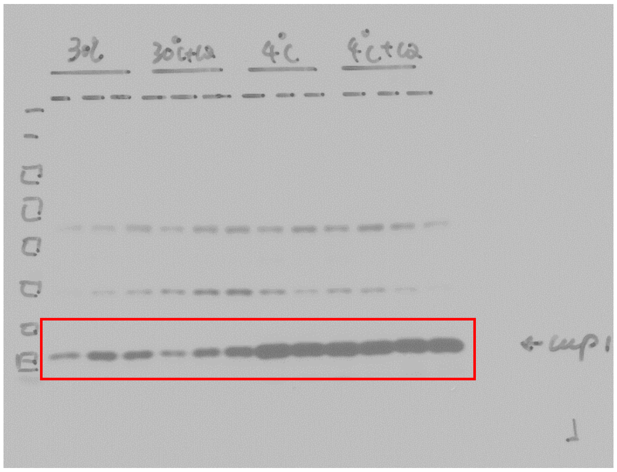

ATP5A

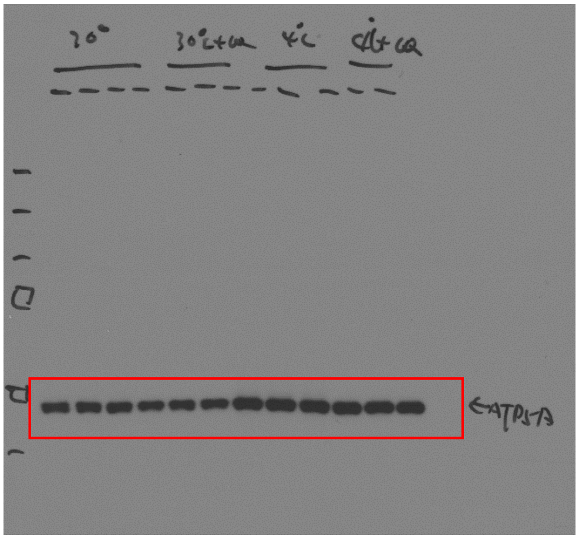

B-actin

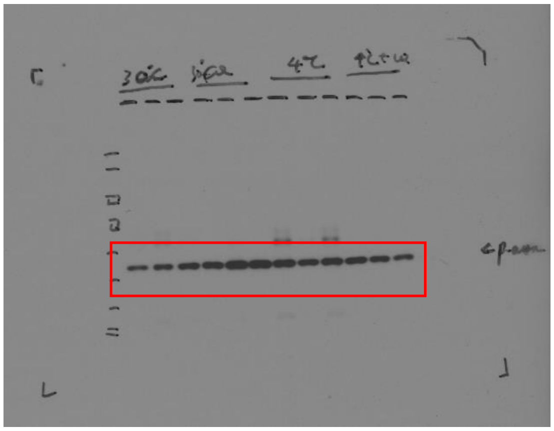

Supplement: Supplementary file 2 — Supplemental upcropped Western Blots [file 41598_2018_26394_MOESM2_ESM.pdf]
